# Supplementary material for: Probing the modulation of enzyme kinetics by multi-temperature, time-resolved serial crystallography
Source: Nat Commun. 2025 Jul 16;16:6553. doi: 10.1038/s41467-025-61631-2 (PMC12267849; doi:10.1038/s41467-025-61631-2)
Supplement: Supplementary file 1 — Supplementary Information [file 41467_2025_61631_MOESM1_ESM.pdf]

Supplementary Information

Probing the modulation of enzyme kinetics by multi-temperature, time-resolved serial crystallography

Eike C. Schulz, Andreas Prester, David von Stetten, Gargi Gore, Caitlin E. Hatton, Kim Bartels, Jan-Philipp Leimkohl, Hendrik Schikora, Helen M. Ginn, Friedjof Tellkamp, Pedram Mehrabi

---

Contents

Supplementary Figures . . . 2

Supplementary Tables . . . 4

  Crystallographic data statistics . . . 4

Supplementary Notes . . . 10

  Environmental control box . . . 10

  Humidity controlled SSX . . . 18

  X-ray induced heating of protein µ-crystals . . . 21

Supplementary Methods . . . 24

  Numerical calculations of the X-ray induced heating of µ-crystals . . . 24

  Characterisation of behaviour of RoPE space plots on number of diffraction patterns. . . . 25

  Group occupancy refinement settings . . . 28

  Electron density figure details . . . 29

Supplementary Code . . . 30

  Script for the shifted inverse gamma distribution . . . 30

  Script to calculate the C_α RMSD . . . 32

  Mathematica 10.4 code for the numerical calculations . . . 35

Supplementary Figures

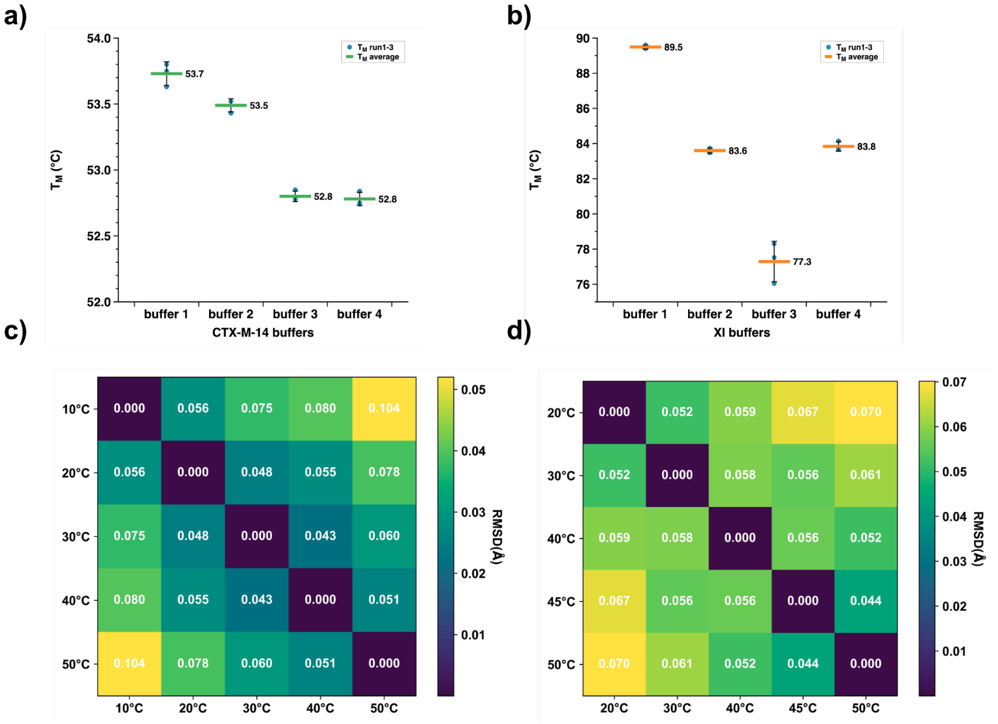

Supplementary Figure 1 In-solution melting temperature reflects the activity optima. a,b) T_{M} determined by nanoDSF ((n=3), with standard-deviation error bars) in 4 different buffer systems (Tab. 1) for each model system, (a) CTX-M-14 green, (b) XI orange. The in-solution melting temperature reflects the activity optima of the mesophilic and hyperthermophilic proteins, respectively. c,d) Pairwise C_α RMSD heatmap. Backbone RMSDs in Å between pairs of structures at different temperatures for both model systems (c) CTX-M-14, (d) XI. Source data are provided as a Source Data file.

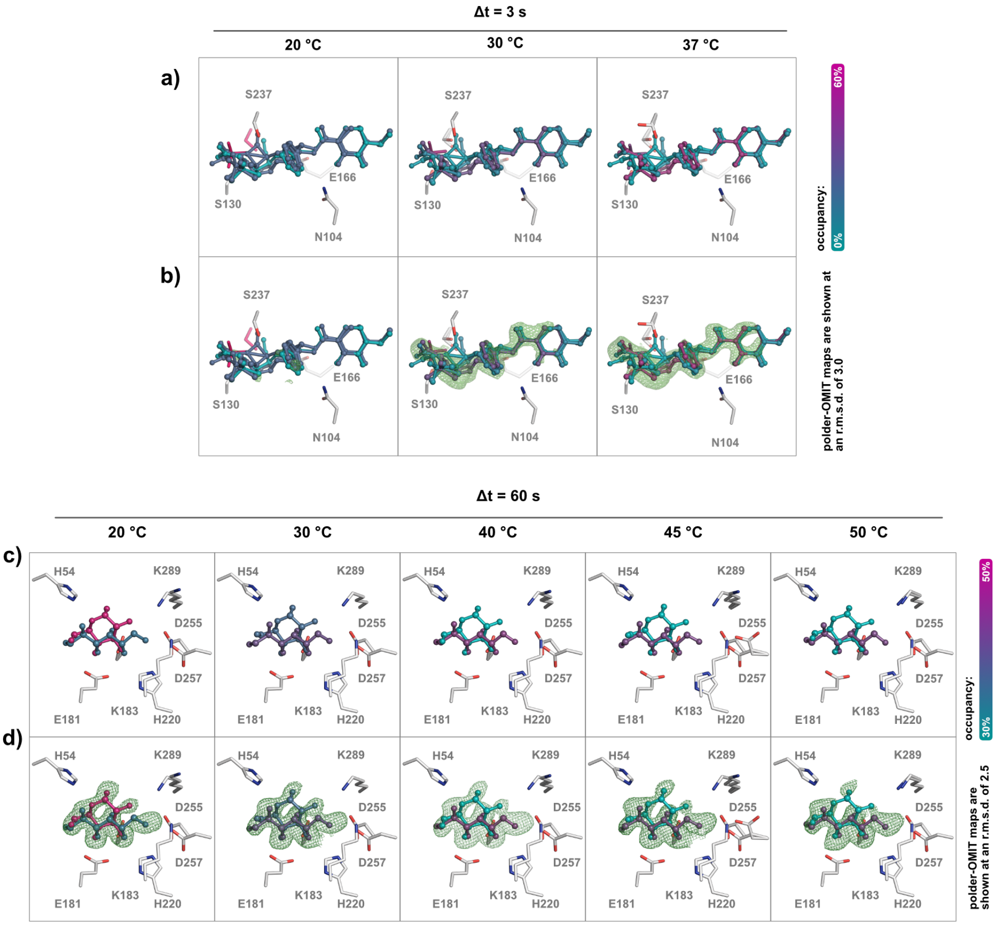

Supplementary Figure 2 Altering enzyme kinetics by temperature modulation. a,b) CTX-M-14 active site, 3 s after reaction initiation at 20 °C, 30 °C, and 37 °C; c,d) XI active site, 60 s after reaction initiation at 20 °C, 30 °C, 40 °C, 45 °C, and 50 °C; a,c) refined fractional occupancy level; b,d) polder-OMIT map shown around the ligands as a green mesh at the indicated RMSD levels.

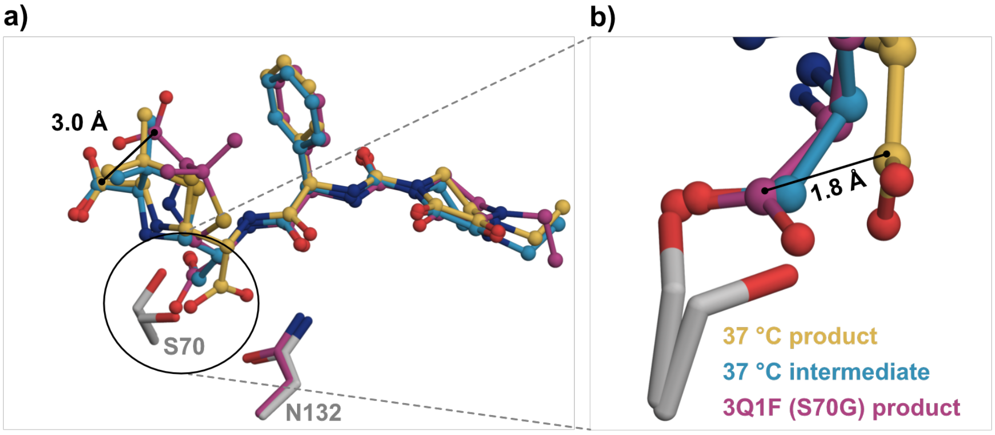

Supplementary Figure 3 5D-SSX reveal new product complex conformation. a,b) CTX-M-14 active site, 3 s after reaction initiation at 37 °C; Comparison to a previously published piperacillin product complex (S70G, PDB-ID: 3Q1F) shows a new orientation of the hydrolysis product in the wild-type protein. Notably, the carboxyl group close to the catalytic Ser70 is shifted by almost 2 Å.

Supplementary Tables

Crystallographic data statistics

Supplementary Table 1 Data collection and refinement statistics for CTX-M-14 apo data. Values in the highest resolution shell are shown in parentheses.

**Temperature (°C) (PDB-ID)**	**10 °C (9G7V)**	**20 °C (9G7W)**	**30 °C (9G7X)**	**40 °C (9G7Y)**	**50 °C (9G7Z)**
Data collection					
Space group	$P322_1$	$P322_1$	$P322_1$	$P322_1$	$P322_1$
Cell dimensions					
a, b, c (Å)	42.23, 42.26, 234.09	42.22, 42.24, 234.15	42.28, 42.31, 234.66	42.32, 42.34, 234.84	42.40, 42.44, 235.33
α, β, γ *(°)*	90.0, 90.0, 120.0	90.0, 90.0, 120.0	90.0, 90.0, 120.0	90.0, 90.0, 120.0	90.0, 90.0, 120.0
Resolution range (Å)	77.62 - 1.70 (1.76 -1.70)	77.62 - 1.70 (1.76 -1.70)	77.62 - 1.70 (1.76 -1.70)	77.62 - 1.70 (1.76 -1.70)	77.62 - 1.70 (1.76 -1.70)
Total reflections	12049024 (713700)	9717308 (575163)	7141365 (422198)	9167246 (544370)	6703777 (398067)
Unique reflections	27454 (2685)	27455 (2685)	27454 (2685)	27454 (2685)	27455 (2685)
Redundancy	428.9 (265.8)	353.9 (214.2)	260.1 (157.2)	333.9 (202.7)	244.2 (148.3)
Completeness (%)	100 (100)	100 (100)	100 (100)	100 (.00)	100 (100)
Mean I/σ (I)	7.99 (1.99)	7.40 (2.17)	6.32 (1.79)	7.02 (1.71)	5.65 (1.04)
Wilson B-factor ($Å^2$)	22.12	21.19	21.97	23.14	25.98
R_{split}	0.101 (0.503)	0.112 (0.453)	0.127 (0.550)	0.112 (0.590)	0.133 (0.962)
CC1/2	0.985 (0.756)	0.979 (0.803)	0.972 (0.723)	0.978 (0.714)	0.967 (0.481)
CC*	0.996 (0.928)	0.995 (0.944)	0.993 (0.916)	0.994 (0.913)	0.992 (0.806)
Refinement					
Reflections used in refinement	27325 (2673)	27341 (2676)	27337 (2671)	27344 (2675)	27322 (2557)
R_{work}	0.1614 (0.2504)	0.1678 (0.2417)	0.1569 (0.2526)	0.1550 (0.2527)	0.1598 (0.3133)
R_{free}	0.1901 (0.2452)	0.1907 (0.2444)	0.1836 (0.2390)	0.1784 (0.2756)	0.1861 (0.2915)
Reflections used for R_{free}	1354 (112)	1354 (111)	1353 (112)	1354 (112)	1353 (111)
Number of non-hydrogen atoms	2242	2230	2209	2196	2177
macromolecules	2067	2060	2046	2044	2037
ligands	5	5	5	5	5
solvent	170	165	158	147	135
Average B-factor ($Å^2$)	28.15	27.03	27.87	28.20	31.46
macromolecules	27.24	26.22	26.97	27.36	30.69
ligands	36.95	34.88	38.77	42.07	46.95
solvent	38.89	36.81	39.20	39.32	42.47
RMS deviations					
Bond lengths (Å)	0.005	0.004	0.005	0.005	0.005
Bond angles (°)	0.772	0.675	0.812	0.825	0.795

Supplementary Table 2 Data collection and refinement statistics of the XI apo data. *Values in the highest resolution shell are shown in parentheses.*

**Temperature (°C) (PDB-ID)**	**20 °C (9G5N)**	**30 °C (9G5S)**	**40 °C (9G5W)**	**45 °C (9G5X)**	**50 °C (9G61)**
Data collection					
Space group	I 2 2 2	I 2 2 2	I 2 2 2	I 2 2 2	I 2 2 2
Cell dimensions					
a, b, c (Å)	94.2 103.05 99.25	94.2 103.05 99.25	94.2 103.05 99.25	94.2 103.05 99.25	94.2 103.05 99.25
α, β, γ (°)	90.0 90.0 90.0	90.0 90.0 90.0	90.0 90.0 90.0	90.0 90.0 90.0	90.0 90.0 90.0
Resolution range (Å)	71.49 - 1.7 (1.761 - 1.7)	71.49 - 1.7 (1.761 - 1.7)	71.49 - 1.7 (1.761 - 1.7)	71.49 - 1.7 (1.761 - 1.7)	71.49 - 1.7 (1.761 - 1.7)
Total reflections	20298653	10229855	10613133	81189855	23289164
Unique reflections	53351 (5284)	53338 (5277)	53317 (5271)	53331 (5273)	53297 (5253)
Redundancy	380.3(224.3)	191.7(113.2)	198.8(117.7)	1521.2(904.5)	436.3(258.8)
Completeness (%)	99.96 (99.96)	99.93 (99.83)	99.89 (99.72)	99.92 (99.75)	99.85 (99.38)
Mean I/σ(I)	5.55(3.31)	3.83(1.50)	3.59(0.98)	8.57(2.88)	4.87(1.14)
Wilson B-factor	15.26	17.54	20.14	19.82	21.96
R_{split}	20.92(33.98)	26.71(66.18)	24.74(102.55)	10.09(31.25)	17.46(90.39)
$CC^{1/2}$	92.76(76.23)	89.39(56.02)	92.27(38.03)	98.64(86.29)	96.25(45.20)
CC*	98.10(93.01)	97.16(84.74)	97.97(74.23)	99.66(96.25)	99.04(78.90)
Refinement					
Reflections used in refinement	53351 (5284)	53338 (5277)	53317 (5271)	53331 (5273)	53297 (5253)
R_{work}	0.1624 (0.2901)	0.1680 (0.3272)	0.1727 (0.3735)	0.1453 (0.3158)	0.1590 (0.3597)
R_{free}	0.1906 (0.3290)	0.2096 (0.3910)	0.2072 (0.4166)	0.1824 (0.3611)	0.1903 (0.3987)
Reflections used for R_{free}	2607 (291)	2607 (291)	2606 (291)	2605 (290)	2603 (288)
Number of non-hydrogen atoms	3637	3566	3638	3552	3543
macromolecules	3275	3234	3325	3261	3284
ligands	2	3	2	3	2
solvent	360	329	311	288	257
Average B-factor ($Å^2$)	17.69	20.2	23.47	22.92	25.36
macromolecules	16.22	19.02	22.33	21.86	24.56
ligands	16.02	11.48	17.73	12.44	18.2
solvent	31.14	31.91	35.64	35.04	35.67
RMS deviations					
Bond lengths (Å)	0.006	0.016	0.004	0.013	0.004
Bond angles (°)	0.8	1.34	0.65	1.23	0.67

Supplementary Table 3 Data collection and refinement statistics of the CTX-M-14 Piperacillin data at a time-delay of 3 s. *Values in the highest resolution shell are shown in parentheses.*

**Temperature (°C) (PDB-ID)**	**20 °C (9G80)**	**30 °C (9G81)**	**37 °C (9G82)**
Data collection			
Space group	$P322_1$	$P322_1$	$P322_1$
Cell dimensions			
a, b, c (Å)	42.15, 42.15, 234.14	42.20, 42.20, 234.53	42.25, 42.25, 234.85
α, β, γ *(°)*	89.99, 89.99, 119.98	89.99, 89.99, 119.93	89.99, 89.98, 119.93
Resolution range (Å)	78.07 - 1.70 (1.76 -1.70)	78.07 - 1.70 (1.76 -1.70)	78.07 - 1.70 (1.76 -1.70)
Total reflections	1266008 (75053)	3599661 (216133)	4883576 (290896)
Unique reflections	27981 (2739)	27439 (2662)	27982 (2734)
Redundancy	45.2 (27.4)	131.2 (81.2)	174.5 (106.4)
Completeness (%)	1.00 (1.00)	1.00 (1.00)	1.00 (1.00)
Mean I/σ (I)	1.91 (0.25)	4.46 (1.04)	4.36 (0.66)
Wilson B-factor ($Å^2$)	26.44	22.25	24.78
R_{split}	0.364 (4.577)	0.182 (0.995)	0.163 (1.595)
CC1/2	0.862 (0.034)	0.952 (0.446)	0.959 (0.252)
CC*	0.962 (0.257)	0.988 (0.786)	0.990 (0.634)
Refinement			
Reflections used in refinement	26826 (1948)	27336 (2363)	27851 (2569)
R_{work}	0.2281 (0.4130)	0.1645 (0.3038)	0.1687 (0.3592)
R_{free}	0.2556 (0.4706)	0.1948 (0.3061)	0.1995 (0.3746)
Reflections used for R_{free}	1315 (103)	1354 (141)	1377 (154)
Number of non-hydrogen atoms	2362	2371	2408
macromolecules	2156	2148	2193
ligands	41	41	41
solvent	165	182	174
Average B-factor ($Å^2$)	36.58	29.28	33.14
macromolecules	35.32	28.21	31.89
ligands	49.55	30.60	39.74
solvent	43.45	40.71	44.02
RMS deviations			
Bond lengths (Å)	0.002	0.005	0.003
Bond angles (°)	0.861	1.046	0.949

Supplementary Table 4 Data collection and refinement statistics of the XI 60 s data. *Values in the highest resolution shell are shown in parentheses.*

**Temperature (°C) (PDB-ID)**	**20 °C (9G6L)**	**30 °C (9G6M)**	**40 °C (9G6N)**	**45 °C (9G6O)**	**50 °C (9G6P)**
Data collection					
**Space group**	I 2 2 2	I 2 2 2	I 2 2 2	I 2 2 2	I 2 2 2
**Cell dimensions**					
a, b, c (Å)	94.2 103.05 99.25	94.2 103.05 99.25	94.2 103.05 99.25	94.2 103.05 99.25	94.2 103.05 99.25
α,β,γ (°)	90.0 90.0 90.0	90.0 90.0 90.0	90.0 90.0 90.0	90.0 90.0 90.0	90.0 90.0 90.0
Resolution range (Å)	71.49 - 1.7 (1.761 - 1.7)	71.49 - 1.7 (1.761 - 1.7)	71.49 - 1.7 (1.761 - 1.7)	71.49 - 1.7 (1.761 - 1.7)	71.49 - 1.7 (1.761 - 1.7)
Total reflections	9415749	12055922	11970298	26069844	8506453
Unique reflections	53357 (5281)	53350 (5281)	53330 (5270)	53342 (5278)	53317 (5273)
Redundancy	176.4(104.5)	225.9(134.2)	224.3(133.2)	488.4(290.4)	159.4(94.7)
Completeness (%)	99.97 (99.91)	99.95 (99.91)	99.92 (99.70)	99.94 (99.85)	99.89 (99.75)
Mean I/σ(I)	4.37(2.68)	4.99(2.46)	4.42(1.65)	6.39(2.67)	3.24(0.93)
Wilson B-factor	15.89	17.92	19.59	21.24	22.4
R_{split}	25.28 (39.40)	20.92 (40.72)	21.34 (60.88)	15.16 (38.29)	27.93 (105.74)
$CC^{1/2}$	89.59(73.39)	92.56(76.39)	93.27(62.3)	96.44(79.42)	89.5(36.2)
CC*	97.22(92.01)	98.05(93.07)	98.24(87.62)	99.09(94.09)	97.19(72.91)
Refinement					
Reflections used in refinement	53357 (5281)	53350 (5281)	53330 (5270)	53342 (5278)	53317 (5273)
Reflections used for R_{free}	2608 (291)	2607 (290)	2605 (289)	2608 (291)	2606 (290)
R_{work}	0.1657 (0.2765)	0.1549 (0.2899)	0.1604 (0.3401)	0.1479 (0.2998)	0.1744 (0.3566)
R_{free}	0.2051 (0.3178)	0.1922 (0.3259)	0.1997 (0.4004)	0.1859 (0.3468)	0.2153 (0.3938)
Number of non-hydrogen atoms	3617	3553	3590	3568	3507
macromolecules	3259	3224	3282	3270	3221
ligands	27	27	27	27	27
solvent	331	302	281	271	259
Average B-factor (Å^2)	19.03	21	23.39	24.18	26.14
macromolecules	17.77	19.93	22.44	23.3	25.37
ligands	14.24	16.09	18.23	19.95	21.63
solvent	31.89	32.89	35.02	35.25	36.23
RMS deviations					
Bond lengths (Å)	0.011	0.014	0.006	0.017	0.006
Bond angles (°)	1.09	1.29	0.8	1.49	0.78

Supplementary Table 5 Data collection and refinement statistics of the XI 180 s data. *Values in the highest resolution shell are shown in parentheses.*

**Temperature (°C) (PDB-ID)**	**20 °C (9I79)**	**50 °C (9I7L)**
Data collection		
Space group	I 2 2 2	I 2 2 2
Resolution range	71.49 - 1.70 (1.76 - 1.70)	71.49 - 1.70 (1.76 - 1.70)
Space group	I 2 2 2	I 2 2 2
Cell dimensions		
a, b, c (Å)	94.20 103.05 99.25	94.20 103.05 99.25
α,β,γ (°)	90.0 90.0 90.0	90.0 90.0 90.0
Total reflections	6213031	5924612
Unique reflections	53348 (5279)	53334 (5270)
Multiplicity	116.4(68.7)	111.0(65.8)
Completeness (%)	99.95 (99.87)	99.92 (99.66)
Mean I/σ(I)	3.99(2.22)	3.38(0.87)
Wilson B-factor	16.45	22.39
R_{split}	29.43(49.09)	25.62(115.22)
$CC^{1/2}$	86.30(62.09)	91.40(33.03)
CC*	96.26(87.53)	97.73(70.37)
Refinement		
Reflections used in refinement	53348 (5279)	53334 (5268)
Reflections used for R_{free}	2607 (291)	2606 (290)
R_{work}	0.1719 (0.2873)	0.1690 (0.3618)
R_{free}	0.2116 (0.3444)	0.2066 (0.4436)
Number of non-hydrogen atoms	3617	3503
macromolecules	3253	3223
ligands	27	27
solvent	337	253
Protein residues	387	386
RMS(bonds)	0.016	0.013
RMS(angles)	1.30	1.22
Average B-factor ($Å^2$)	18.94	25.78
macromolecules	17.73	25.09
ligands	16.09	22.01
solvent	30.85	34.99

Supplementary Notes

Environmental control box

Design

To maintain a controlled humidity environment for our hit-and-return (HARE) chip setup including the liquid-application-method for time-resolved crystallography (LAMA) [40, 42], required the development of a solution that could accommodate this experimental setup. To this end we have constructed a modular, compact environmental control box that encloses our previously described chip setup including the LAMA droplet injector nozzle on a footprint of 118 mm x 283 mm (Sup. Fig. 4, 5) [43]. Humidity control is achieved by flowing dry air either directly into the box, or first passing through a water bath (20-95 °C), with the proportion of gas through each channel controlled by a toggling ball valve in a proportional-integral-derivative (PID) feedback loop. The water bath is connected to the environmental control box via a silicone hose, to which a heating belt is attached preventing condensation. The set point can be achieved with high accuracy, enabling humidity control within 1 percent point of relative humidity, which allows to perform controlled crystal dehydration if that is required. Many beamlines are equipped with a temperature control solution via a gas stream that is directed at the sample, sometimes encompassing wide temperature windows but typically limited to room temperature. Interestingly, however, a combined temperature-humidity control is rarely found. Such a situation mandates the use of e.g., glass capillaries to maintain the crystals in a humid environment, which complicates time-resolved applications that are based on in-situ mixing. Historically, flow-cells were developed for time-resolved applications that allowed for in-situ mixing experiments with single crystals and for trapping reaction intermediates on comparably slow time scales [85–87], which were recently extended to SSX experiments [88]. However, to the best of our knowledge these were not applied to multi-temperature experiments. Serial crystallography experiments can also be conducted in controlled,

closed-boundary environments as demonstrated by the drop-on-demand device, which permits humidity control and fully anaerobic experiments via the exchange of the surrounding atmosphere [89]. In addition to humidity control, the temperature within our enclosure can be adjusted anywhere within the range of approximately 7 °C to above 70 °C. An air-stream reflector directs the stream of humid air around the chip. In order to enable effective control over this wide temperature range, the box uses two interchangeable modular temperature control units (Sup. Fig. 5). Module-1 covers a temperature range from approximately +7 °C to approximately +50 °C, while module-2 covers a temperature range from approximately +50 °C to over +70 °C. Rapid exchange of the modules is possible without tools, enabling switching between different temperature regimes within a few minutes. Module-1 contains water-cooled Peltier-elements that enable active cooling or heating of the interior of the box. To ensure that the temperature is equilibrated across the box, the module is equipped with fans. Cooling water and electric power are fed in through the top side of the module. The heating element in module-2 is a power resistor network that disseminates sufficient heat to increase the interior temperature of the box to over 70 °C, while the relative humidity can be sustained at over 95%. Temperature control is achieved by a PID controller that sets the current through the Peltier elements and resistor network, respectively, to maintain the target temperature. The base plate of the box is made from durable polyether ether ketone (PEEK). To drain condensation water, several cotton wicks are fixed around the bottom corners and connected to an active pumping system that quickly drains excess liquid from the box. The sides and the lid of the box are made of 6 mm thick acrylic glass, while the rear panel (X-ray side) is made of acrylic glass and polyoxymethylene (POM). As an X-ray entrance window, an 8 mm opening in the POM is covered with two spaced layers of COC foil, about 1 mm apart. To enable easy replacement of the X-ray entrance window, the COC foil is placed on a magnetically mounted ring that tightly seals the inside of the box. While one side

panel is solid, the other side panel has a feedthrough for the humidity tube, and an access hatch through which HARE chips can be loaded onto the sample translation stage. The front panel (detector side) is an aluminum frame with a 190 mm opening. This is sealed with two X-ray transparent Mylar foils (6 µm), as an exit window for the diffracted beam. To reduce condensation on this window, the space between the Mylar foils is continuously flushed with warm air. The lid of the box contains feedthroughs for the humidity and temperature sensors, as well as for the electropneumatic retractable infrared (IR) backlight, the LAMA-nozzle lever, and an access port for heating module exchange. The translation stage system implemented at the T-REXX endstation is not humidity resistant and therefore has to be kept outside of the box. Hence, the translation stages are connected to the box via a flexible bellow, custom cut from two layers of commercial plastic wrap. On the inside, the bellow is sealed between the translation stages and the kinematic mount for the chip holder [63, 90]. To reduce the heat capacity of the chip holder and thus achieve faster temperature equilibration, the previously described aluminum chip holder was redesigned from PEEK, providing the same functionality at a lighter weight [63]. The LAMA nozzle is attached *via* a kinematic mount to a retractable lever that enables retraction of the nozzle from its injection position during chip exchange. To avoid the unnecessary opening of the box, which might lead to temperature and humidity fluctuations, the nozzle retracts into a parking position under the lid of the box. Fine alignment of the LAMA nozzle in the injector position is achieved *via* motorized translation stages (Thorlabs). The whole box system is mounted on rails, residing on a stainless-steel baseplate that can be moved between “data collection” or “beam location” position, where the latter allows to use the X-ray scintillator built into the beam-shaping device (BSD; Arinax, Moirans, France). Operation of the serial crystallography environmental control enclosure is achieved *via* an external control unit where temperature and

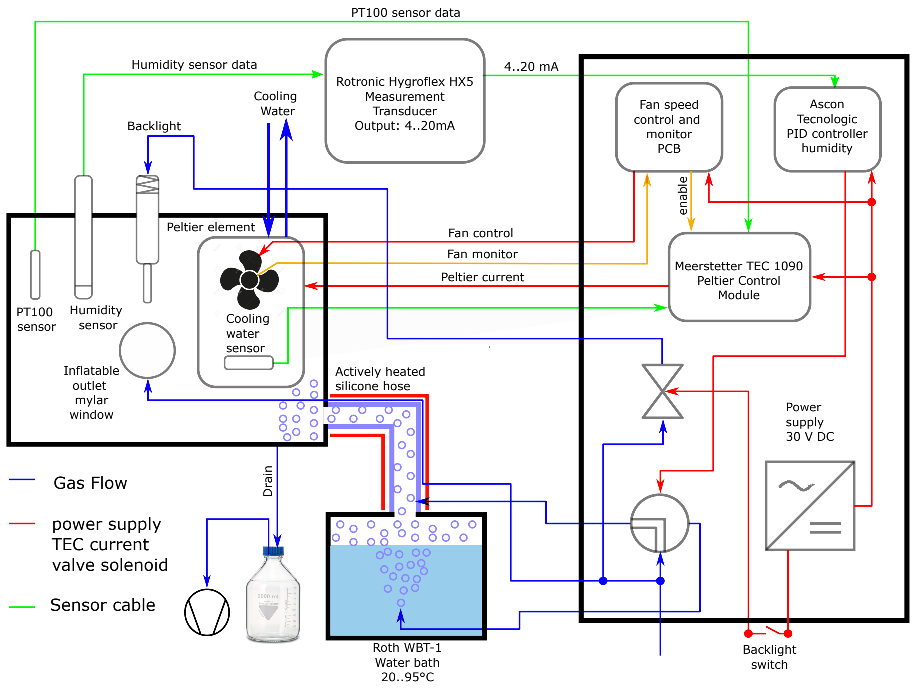

Supplementary Figure 4 Schematic representation of the environmental control box setup. Gas flow is indicated by blue lines, power supply is red, sensor cables are depicted in green, and cooling water is displayed in pink.

humidity parameters are electronically set (Sup. Fig. 6). Parameters needed to achieve certain conditions were calibrated and are listed in **Sup. Tab. 6**.

Characterization of temperature and humidity stability

To characterize how the environmental parameters can be controlled within the box, we recorded temperature and humidity changes as a function of time in 30 s intervals. The temperature was modified as a step function, with the humidity target set to 95%. In addition to the overall temperature inside the box, measured near the panel opposite to the access hatch, we also monitored the temperature at two additional locations, directly on the surface of the backside of the HARE chip and directly above the humidity sensor (Sup. Fig. 7). To assess how effectively different temperatures in the

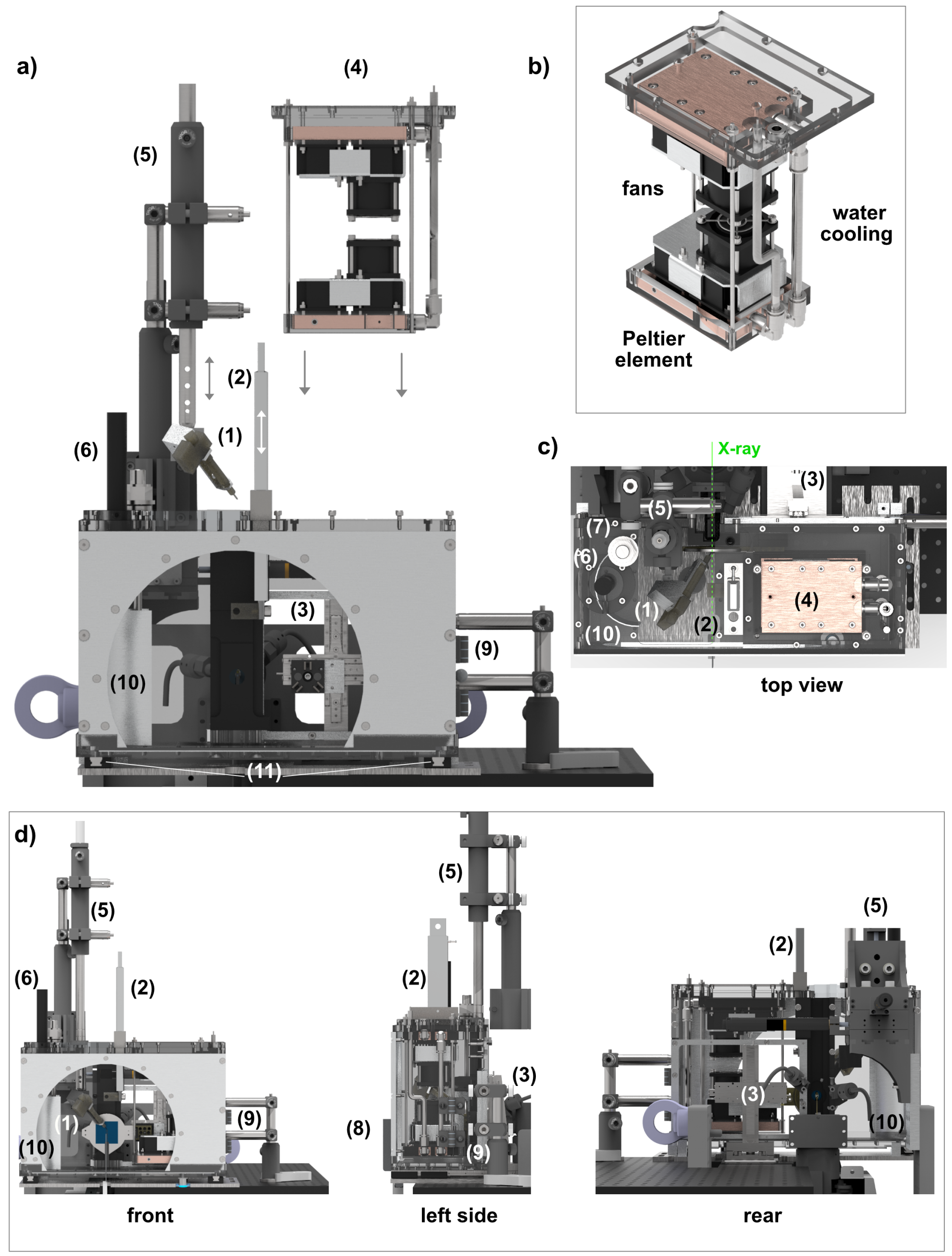

(1): LAMA nozzle **(4): Peltier module** **(7): temperature sensor** **(10): air stream reflector**

(2): IR backlight **(5): LAMA guillotine** **(8): beamstop** **(11): retraction rails**

(3): translation stages **(6): humidity sensor** **(9): access hatch**

Supplementary Figure 5 The Serial Crystallography Environmental Control Box. a) overview of the environmental control box, with retracted LAMA nozzle and Peltier module-1. b) closeup of the Peltier module-1, c) top-view providing an overview of the arrangement inside the box, d) front-, side- and rear-view of the box. Note: for clarity module-2 and some technical elements of the box (e.g. tubing, electric connections etc.) or the beamline are not shown or described in detail. Elements mentioned in the text are numbered and shown in the figure legend.

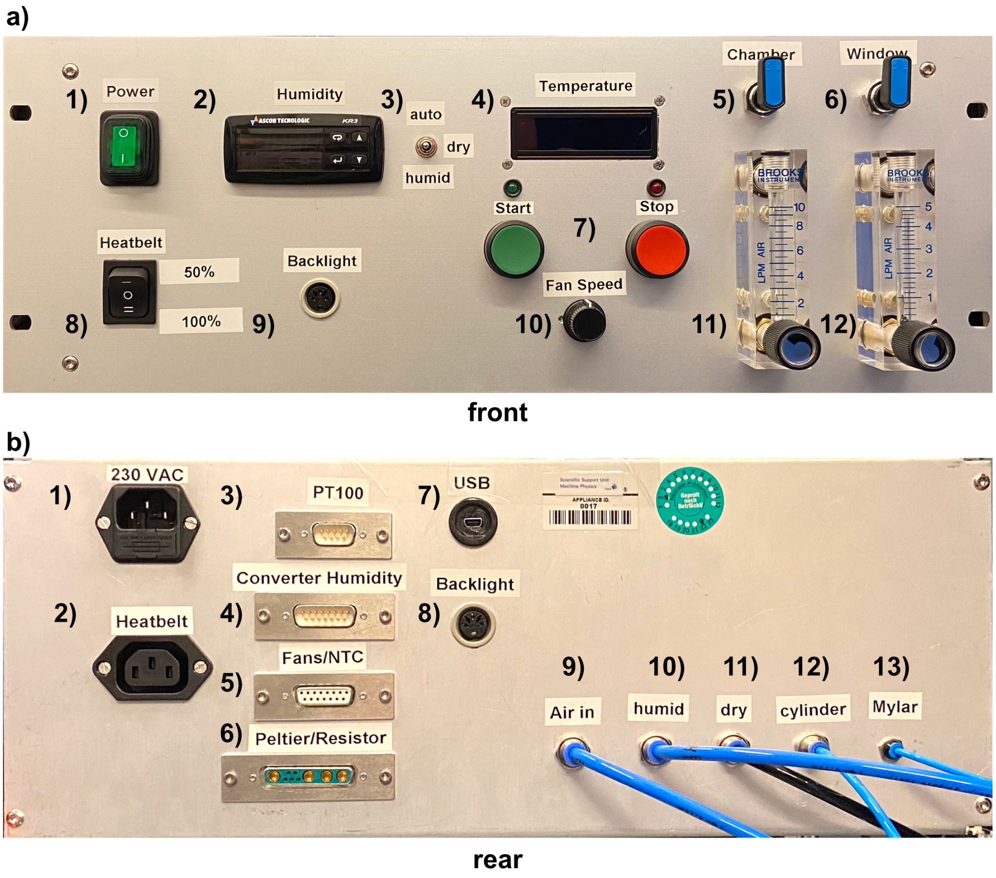

Supplementary Figure 6 Control unit of the serial crystallography environmental control box. a) front panel: 1) main power switch; 2) humidity control/display; 3) air flow switch; 4) temperature display; 5) airflow on/off switch; 6) inflatable front-window flow on/off switch; 7) Temperature control start/stop button; 8) heat-belt switch; 9) socket for IR backlight switch; 10) fan-speed regulator; 11) air-flow valve; 12) front-window flow valve. b) rear panel: 1) main power inlet; 2) heat belt power outlet; 3) PT100 temperature sensor; 4) humidity sensor; 5) fan connection; 6) Peltier/resistor connection; 7) USB connection to PC; 8) backlight connection; 9) pressurized air in; 10) air flow to water bath; 11) dry air outlet; 12) backlight cylinder air outlet; 13) inflatable front-window air outlet.

box can be achieved and maintained, we characterised the temperature increase from 7.5 °C to 80 °C. The data show that for both temperature control modules the humidity values quickly reach the target values. Over a temperature window of ca. 70 °C the humidity remains stable within 2.5% of the set point. Analysis of the deviation of the chip temperature from the box temperature shows that the chip temperature follows the box temperature with a median difference of 0.7 °C, over a temperature window of

Supplementary Table 6 Environmental control parameter settings

	desired temp. °C	flow rate L min^{-1}	desired humidity (%)	actual humidity (%)	water bath temp °C	heat belt power (%)	module current (A)
module-1	7.5	5	95	95	75	0	-12.3
	15	5	95	95	75	50	-2.1
	25	5	95	95	75	100	1.7
	35	4	95	95	85	100	3.5
	45	4	95	95	95	100	4.8
	55	4	95	95	95	100	6
module-2	50	4	95	95	95	100	2.4
	55	4	95	95	95	100	2.9
	60	4	95	95	95	100	2.6
	65	4	95	95	95	100	2.9
	70	4	95	95	95	100	3.1
	75	4	95	95	95	100	4.3
	80	4	95	95	95	100	3.4

ca. 70 °C. Temperature and humidity typically equilibrate across the box and the chip within 10-15 minutes. We also examined the reliability of maintaining environmental set points during X-ray data collection. To this end we collected X-ray diffraction data at 20 °C, 40 °C, 55 °C and 80 °C, for ca. 120 minutes and recorded temperature and humidity values in 30 s intervals during this period (Sup. Tab. 7). Remarkably, during the data collection the target humidity could be maintained within approximately 1%, while the temperature remained stable within approximately 0.5 °C. Clear deviations from this behavior are only observed during chip exchange, when the hatch of the box is opened and during the subsequent re-equilibration time while the environment stabilises. Re-equilibration of the environmental parameters could be achieved within 10-15 minutes, depending on the duration of the manual chip exchange. In conclusion, these data show that after an equilibration time of approximately 10-15 minutes the environment in the control box has reached its target value, and can be maintained throughout extended periods of time, well beyond the typical data collection time of a chip (ca. 30 minutes). This enables collection of serial X-ray diffraction data at a variety of different temperature and humidity levels with high accuracy and precision.

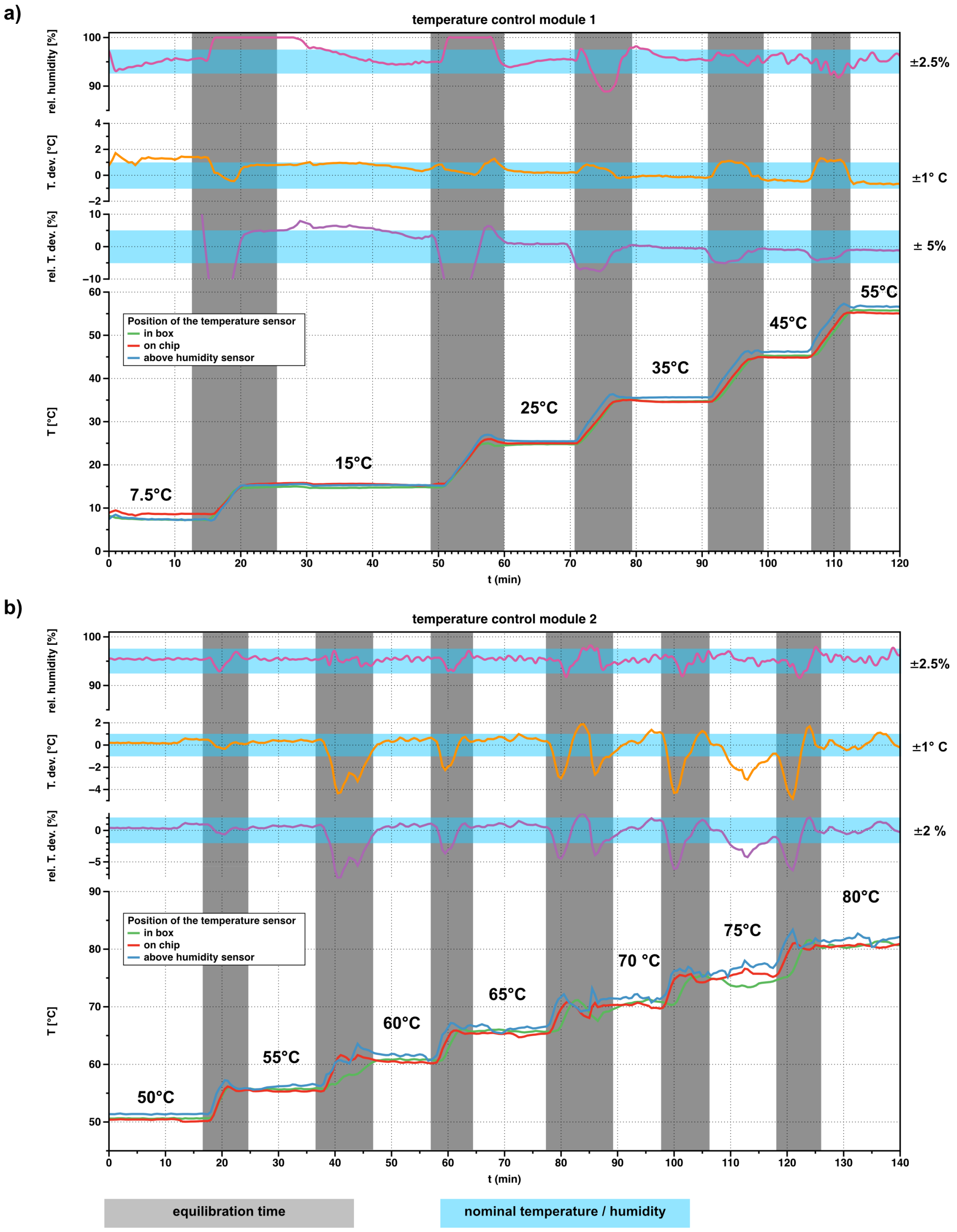

Supplementary Figure 7 Characterization of the environmental control. a) module-1: 7.5 °C – 55 °C; b) module-2: 50-80 °C; The temperature was successively increased from 7.5 °C to 55 °C and from 50 °C to 80 °C respectively. The temperature was measured at three positions inside of the chamber: green inside the box near the panel opposite to the access hatch, blue directly above the humidity sensor, red directly on the chip. The target humidity was set to 95%. The grey bars indicate the equilibration time, blue bars indicate the target window.

Supplementary Table 7 Environmental parameters during X-ray diffraction data collection. *values are derived from a long-term (10 h) experiment

nominal temp. (°C)	nominal rel. humidity (%)	measured rel. humidity (%)	RMSD (%)	measured temp. (°C)	RMSD (°C)
20	95	95.4	0.5	19.8	0.2
40	95	95.5	0.9	40.3	0.3
55	95	95.5	0.7	55.3	0.7
80	95	95.2	0.7	80.1	0.3
20*	95*	95.2*	0.7*	19.8*	0.2*

Humidity controlled SSX

A hallmark of protein crystals is their large solvent content, which is typically in the range between 40% and 70% of the crystal volume. An advantage of this property is that proteins generally retain their function even in the crystalline state [91, 92]. A commonly known disadvantage is, however, their sensitivity to changes in environmental humidity, which in addition to the higher rates of radiation damage associated with higher temperatures makes routine data collection at even ambient temperatures a difficult task. Accordingly, starting with traditional wax-enclosed glass capillaries, environmental control has consistently been a key aspect of macromolecular crystallography, and several solutions have been developed to maintain crystal hydration for single crystals [4, 93]. Open- and closed boundary environmental control solutions have been developed since the advent of structural biology. In the simplest instances, closed boundary devices include glass capillaries, which contain protein crystals and typically a drop of mother liquor to sustain a humid atmosphere during data collection [93]. More modern variations of this classic solution are the many fixed-target serial crystallography environments, which protect protein microcrystals against evaporation by some form of X-ray transparent window material [94]. For single crystal experiments a variety of solutions, sometimes for advanced parameter control, such as humidity, temperature, and electric fields have also been developed over the years [95–97]. With the onset of cryo-crystallography, larger boxes were developed, which sometimes enclosed

the stream of cryogenic gas in a dry atmosphere to prevent ice-formation during data-collection [98]. However, for single-crystal experiments the majority of environmental control solutions fall into the open-boundary category, such as placing the crystal in an vapor stream with controlled humidity [36, 96, 99–102]. Historically, the water content of protein crystals was controlled by post-crystallization treatments via chemical dehydration prior to crystal freezing [34, 103]. However, controlling the humidity around the mounted crystals enables the convenient identification of the optimal conditions for a particular sample during an X-ray diffraction experiment [36, 96, 99–102]. Adjusting the relative humidity either prior to, or during data collection can improve several aspects of data quality (resolution, mosaicity and anisotropy) [36, 37, 39, 99, 101, 104–107]. To estimate the effect of the environmental humidity on diffraction data quality we monitored the unit-cell size of XI as a function of decreasing humidity. We started data collection at a relative humidity of 95% and reduced the humidity in steps of 5% per compartment row on the chip (**Fig. 8**). With decreasing humidity the crystals do not simply cease to diffract but undergo a change in unit-cell size. While at or above a relative humidity of 95% most diffraction patterns could be indexed with a larger unit-cell (a = 94.2 Å, b = 99.3 Å, c = 103.1 Å; $\alpha, \beta, \gamma = 90.0°$), but the proportion rapidly changed to a smaller unit cell (a = 94.6 Å, b = 99.4 Å, c = 87.5 Å; $\alpha, \beta, \gamma = 90.0°$) as the relative humidity dropped from 90% to 75%. If the humidity is reduced even further the micro-crystals cease to diffract, presumably due to complete dehydration. This emphasizes the sensitivity of protein micro-crystals to environmental humidity, which must be precisely controlled to maintain their diffraction properties. On the other hand, this also opens the opportunity for crystals with large unit cells to be specifically dehydrated to modulate their diffraction properties. The response of protein crystals hydration to their environment has long been known [34] and chemical dehydration devices [103, 108–110] as well as dedicated de-humidification devices have successfully been used for this purpose on single, loop-mounted crystals [39, 99, 101].

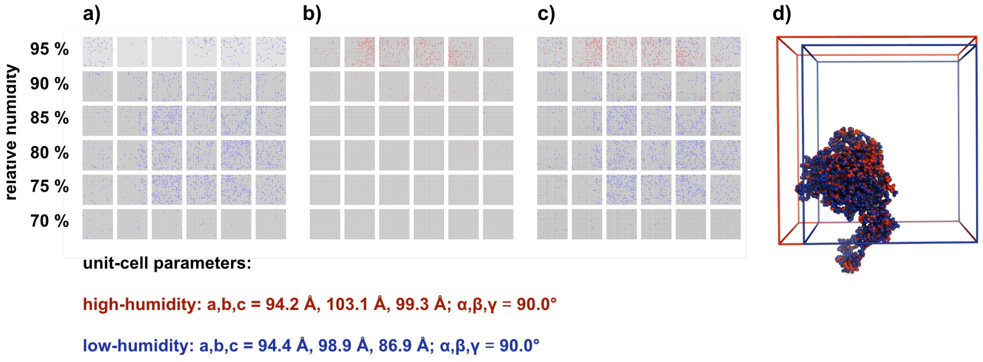

Supplementary Figure 8 Humidity dependent unit-cell modulation displayed in a hit map. The HARE chips consist of 6x6 compartments, each divided into 24x24 features. Each feature on the HARE-chip that can contain a crystal is represented by a small grey square; if a diffraction pattern is recorded the particular square is highlighted in colour. A blue square indicates a diffraction pattern corresponding to a low humidity unit cell, while a red square corresponds to a diffraction pattern in the larger high-humidity unit cell. Humidity has been reduced by 5% for each row of compartments. a) recorded diffraction patterns corresponding to low-humidity unit-cells b) recorded diffraction patterns corresponding to high-humidity unit-cells; c) overlay of low- and high-humidity unit-cell diffraction pattern hits; d) comparison of high- and low-humidity unit-cells.

With our environmental control box these post-crystallization optimization protocols are now open to serial crystallography.

X-ray induced heating of protein µ-crystals

While the environmental control box provides a stable temperature bath for the protein crystals, their temperature is also altered by the incident X-ray beams during data collection. As laid out by Warren et al. [111] the simplest estimate of beam-induced temperature changes is provided by an adiabatic model that ignores any heat-exchange with the environment. Thus the maximum possible increase in temperature is given by the energy absorbed by the mass and the specific heat capacity of the sample. Since the energy absorbed by the mass can also be expressed by the absorbed dose, this can be expressed as:

$$\Delta T = \frac{Q}{m \cdot c_\rho} = \frac{Q_D}{c_\rho} \tag{1}$$

,wherein ΔT is the temperature change, $\frac{Q_D}{m}$ is the energy absorbed per unit mass, c_ρ is the specific heat capacity, and Q_D is the absorbed dose. The specific heat capacity of protein crystals is approximated via those described for tetragonal lysozyme (1.8×10^3 J/ kg K) [112]. The absorbed dose was calculated using *RADDOSE-3D* [113], based on the parameters given in Table 8, below.

However, as an adiabatic model clearly ignores heat-exchange with the mother-liquor, to the crystalline silicon-chips, to the air-interface inside the wells, as well as convectional cooling, photoelectron escape or any energy conversion by radiolytic processes, we consider the adiabatic temperature increase an upper boundary, while realistic temperature changes are certainly well below these values. In order to further investigate this, we have modelled the thermal diffusion process into the surrounding regions using numerical simulations (Suppl. Methods). To predict the spread of heat, if thermal diffusion is taken into consideration we employed a conservative model. To

Supplementary Table 8 Raddose-3D parameters to calculate the upper boundary of X-ray induced heating.

**Protein**	**XI**	**CTX-M-14**
Crystal type	Cuboid	
Dimensions [µm]	20, 20, 10	15, 15, 10
Beam type	Gaussian	
Flux [ph/s]	1.00×10^{12}	1.10×10^{12}
FWHM [µm]	30, 10	30, 10
Energy [keV]	12.699	12.699
Collimation Rectangular [µm]	10, 9	10, 9
Wedge	0, 0.0001	0, 0.0001
Exposure time [s]	0.007	0.005
Average Dose (exposed region) [J/kg]	36222	20234
specific heat capacity [J/kg K]	1.8 x 1.8×10^3	1.8×10^3
adiabatic model ΔT [K]	20.12	11.24
diffusive model ΔT [K]	0.7	0.5

this end we utilised the thermal diffusivity of water ($\alpha = 1.4 \times 10^{-6} mm^2/2$) as a lower-bound estimate, compared to crystalline environments (Sup. Tab. 9).

Supplementary Table 9 Parameters used to calculate the X-ray induced heating in protein mirco-crystals.

**Abbreviation**	**Quantity**	**Units**	**Reference**
c_ρ	Heat capacity for tetragonal lysozyme	1.8×10^3 J / kg $\cdot K$	Kriminski et al 2003 [112]
α	Thermal diffusivity of water	1.4×10^6 mm^2 / s	Blumm et al 2003 [114]

The simulations, which include the diffusion of heat to the surrounding region, therefore exhibit an over-estimation of the possible temperature increase. Considering this more realistic scenario with heat dissipation into the surrounding region, yields a temperature increase of 0.7 K for XI and 0.5 K for CTX-M-14, respectively (Sup. Fig. 9; Sup. Tab. 9). However, with respect to turnover kinetics the delay time after reaction initiation has to be taken into consideration. While the X-ray exposure time was 5 and 7 ms, respectively, the delay times were orders of magnitude larger for both CTX-M-14 (3s), and XI (60s, 180s). This long time delay enables progression of ligand binding and turnover kinetics, irrespective of any potential X-ray induced heating. While we note that this temperature increase of 0.5°- 1°C may further influence the

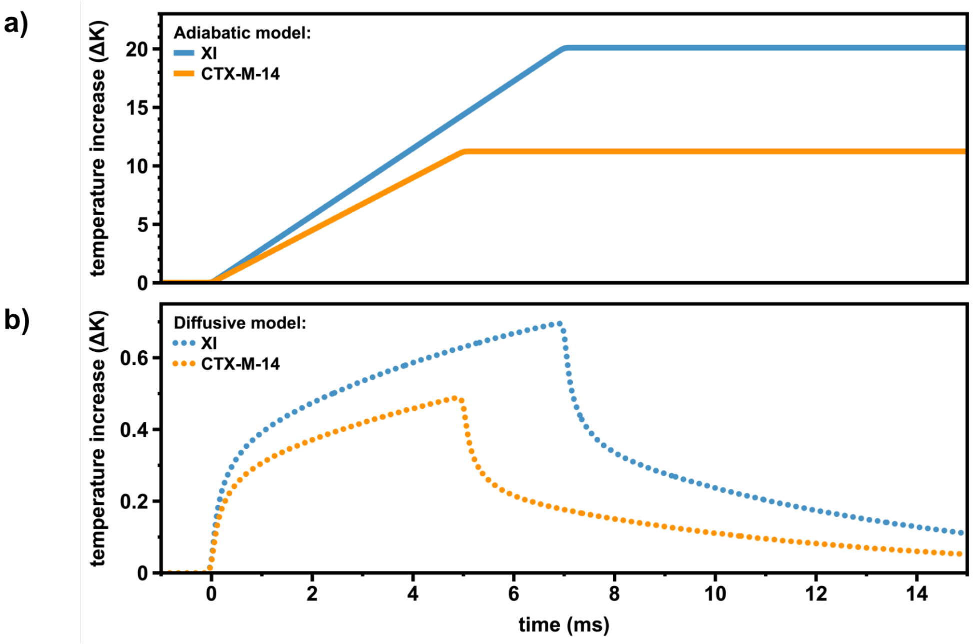

Supplementary Figure 9 X-ray induced heating in protein μ-crystals. (upper panel - adiabatic model): A temperature increase of up to 20K can be observed if no heat dissipation into the surrounding medium is considered. (lower panel - diffusive model): However, if the surrounding region is thermally conductive the temperature increase is limited to less than 1K, for each case.Source data are provided as a Source Data file.

turnover kinetics, it is important to emphasize that this upper boundary can be considered a constant offset that applies to all time-points. That is the deviation from the nominal temperature change would affect all time- and temperature-points uniformly, and therefore not lead to deviations from the relative temperature changes set by the environmental control box.

Supplementary Methods

Numerical calculations of the X-ray induced heating of µ-crystals

Calculations were made in order to approximately determine the temperature increase caused by the absorption of the X-ray beam. The absorbed dose Q_D was first determined using RADDOSE-3D [113], based on a static model without thermal diffusion (see below). The temperature rise ΔT is then determined by $\Delta T = \frac{Q_D}{c}$, where c is the specific heat capacity.

If thermal diffusion is taken into account, the resulting temperature increase is lower, as the absorbed energy spreads into the surrounding regions. To quantify this effect, numerical simulations were performed using *Mathematica* [115]. For the details of the Mathematica-code, please refer to the code-section below.

The heat equation $\dot{u} = \Delta u$ was applied, restricted to the two spatial dimensions perpendicular to the beam axis. Additionally, a time- and position-dependent source term, $R \cdot Q_\mathrm{S}(\mathbf{r}, t)$, was included to represent the heat input from the X-ray beam. This beam was modelled as a collimated Gaussian profile $Q_\mathrm{S}(\mathbf{r})$ active only during the exposure time t_exp (Sup. Fig. 10).

$$\partial_t u(\mathbf{r}, t) = \alpha \ \Delta u(\mathbf{r}, t) \ + \ R \cdot Q_\mathrm{S}(\mathbf{r}, t), \quad \text{with} \tag{2}$$

$$Q_\mathrm{S}(\mathbf{r}, t) = \left| \begin{array}{ll} Q_\mathrm{S}(\mathbf{r}) & \text{if } 0 \le t \le t_\mathrm{exp} \\ 0 & \text{otherwise} \end{array} \right. \tag{3}$$

The parameter R was chosen so that the temperature increase when heat diffusion is neglected and thermal diffusivity α is zero, matches the results obtained from RADDOSE-3D, thus $R = \frac{\Delta T}{t_\mathrm{exp}}$. Enabling thermal diffusion by assigning α a non-vanishing value results in a temperature increase of less than 1 K, compared to roughly 20 K from calculations without diffusion.

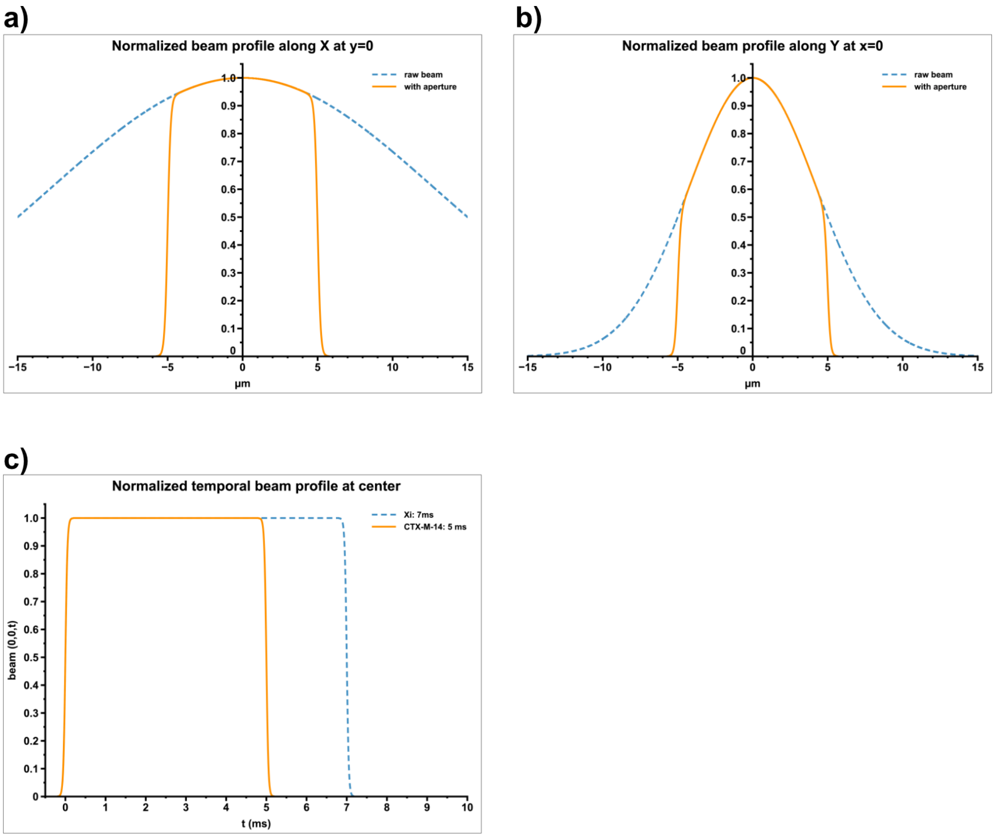

Supplementary Figure 10 Beam profile used in numerical calculations for the X-ray induces heating. a) normalized beam profile along X at y=0; b) normalized beam profile along Y at x=0; c) normalized temporal beam profile at the centre. Source data are provided as a Source Data file.

Characterisation of behaviour of RoPE space plots on number of diffraction patterns.

RoPE space plots take advantage of the oversampling of serial diffraction data in order to establish a statistical support for interpretation of structural differences, by splitting the data for independent structure solutions. This needs to strike a balance, for a given number of diffraction patterns, between the total number of structures and the number of diffraction patterns assigned to each structure. The number of diffraction patterns will influence the quality of the data supporting the analysis. Here

we vary the approximate number of diffraction patterns in each structure from 3000 to 8000 images, in steps of 1000 images. Naturally, with more images per structure, fewer structures are generated. We see that the separation is present for all image numbers per structure (Sup. Fig. 11 a), but to some extent, the ”tightness” of each cluster increases with more supporting images per structure. Showing all structures at the same time, this time coloured by number of images (Sup. Fig. 11 b), the clusters corresponding to respective temperatures are still recognisable. However, this shows that structures supported by fewer images are more similar to each other despite being different temperatures. This is likely because of poorer quality diffraction intensity estimates and greater reliance on the geometry term rather than the X-ray data during refinement. However, structures supported by more images can reveal more features associated with the given temperature, due to higher quality intensity estimates, and therefore are spaced further apart from one another.

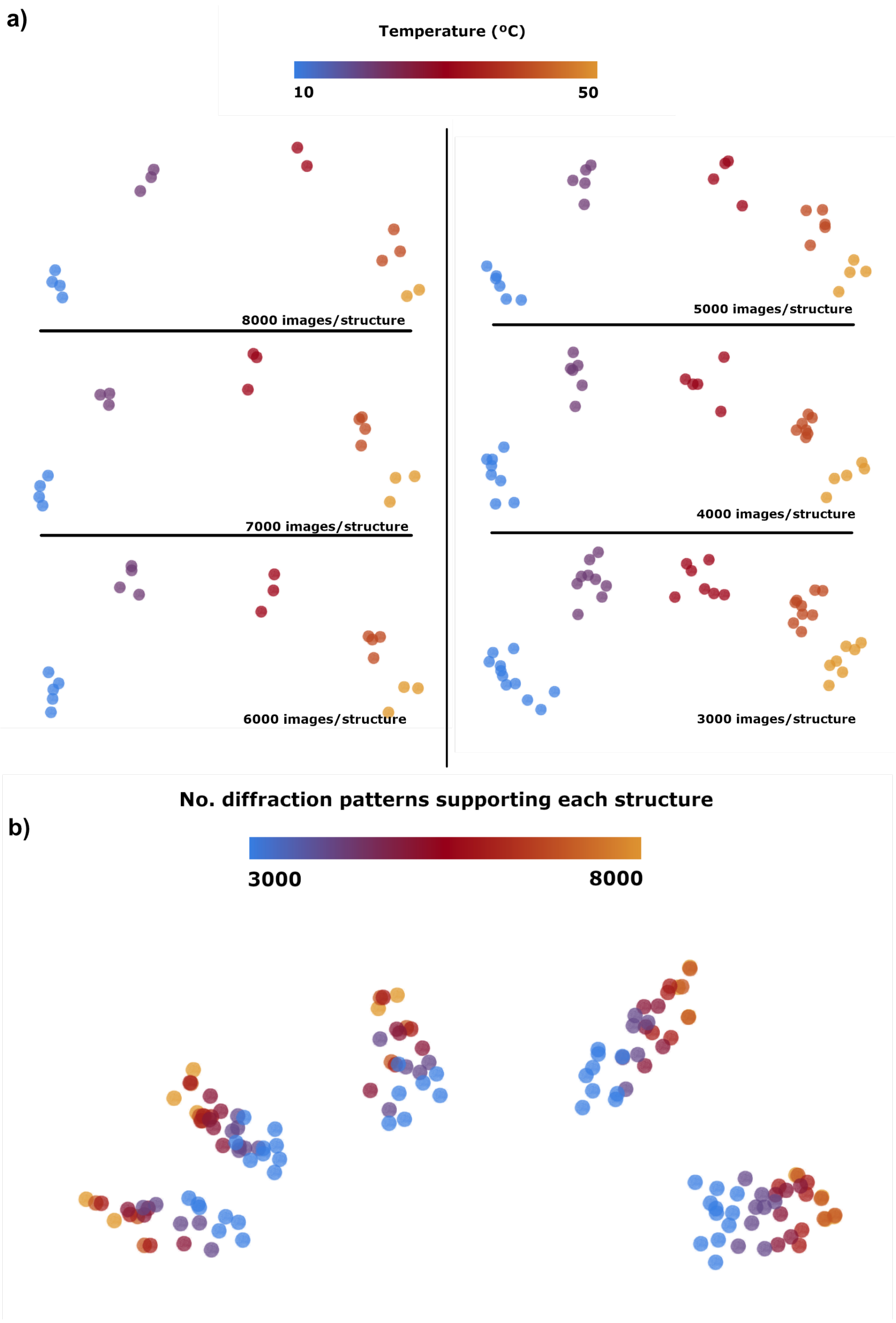

Supplementary Figure 11 Titration of the number of diffraction patterns for ROPE analysis. a) Titration of successively lower number of diffraction patterns per structure used to generate the RoPE space. b) RoPE space summarising the behaviour of varying the number of diffraction patterns supporting each structure.

Group occupancy refinement settings

Following group occupancy refinement setting have been used for CTX-M-14:

```
occupancies {
    individual = None
    remove_selection = None

    constrained_group {
        selection = resseq 301 and chain A and altloc A
        selection = resseq 302 and chain A and altloc B
        selection = resseq 302 and chain A and altloc C or resseq 70 and chain A and altloc C
        selection = resseq 162 and chain S or resseq 163 and chain S or resseq 164 and chain S or resseq 165 and chain S or resseq 167 and chain S or resseq 168 and chain S or resseq 171 and chain S or resseq 173 and chain S or resseq 174 and chain S or resseq 176 and chain S or resseq 177 and chain S or resseq 180 and chain S or resseq 181 and chain S or resseq 1 and chain B
    }
    constrained_group {
        selection = resseq 70 and chain A and altloc C
        selection = resseq 70 and chain A and altloc A
      }
}
```

Following group occupancy refinement setting have been used for XI:

```
occupancies {
      individual = None
      remove_selection = None
      constrained_group {
        selection = chain C and element Mg
      }
      constrained_group {
        selection = chain D and element Mg
      }
      constrained_group {
        selection = chain A and resseq 402 and resname HOH or chain A and \
                    resseq 403 and resname HOH or chain A and resseq 404 and \
                    resname HOH or chain A and resseq 405 and resname HOH or \
                    chain A and resseq 406 and resname HOH or chain A and \
                    resseq 407 and resname HOH
        selection = chain A and resseq 401 and resname GLO
```

```
        selection = chain A and resseq 401 and resname GLC
    }
    }
```

Electron density figure details

Supplementary Table 10 PyMol color settings to display volume elements.

Color	map RMSD.	R	G	B
Blue	1.0	19	43	157
Cyan	1.5	153	219	223
Green	2.0	101	198	66
Yellow	2.5	255	240	0
Orange	3.0	252	131	0
Red	3.5	245	38	0

Supplementary Code

Script for the shifted inverse gamma distribution

```python
#!/usr/bin/env python3
# -*- coding: utf-8 -*-
"""
Created on Wed Jul 17 10:08:46 2024

@author: gargi
"""

import numpy as np
import matplotlib.pyplot as plt
from scipy.special import gamma
from scipy.optimize import curve_fit

# Define a Shifted Inverse Gamma Function
def sigd(x, a, b, d):
    return (b**a / gamma(a)) * ((x - d)**(-a - 1)) * (1 / np.exp(b / (x - d)))

# Common settings for plots
plt.rcParams['figure.dpi'] = 300
plt.rcParams['savefig.dpi'] = 300

# File paths and colors
file_paths = ['','','','','','']


colors = ['royalblue', 'darkviolet', 'limegreen', 'gold', 'orangered']
labels = ['10 C ', '20 C ', '30 C ', '40 C ', '50 C ']

all_fits = []

for idx, (file_path, color, label) in enumerate(zip(file_paths, colors, labels)):
    b_values = np.loadtxt(file_path, dtype=float)
    freq, bins, _ = plt.hist(b_values, bins=80, density=True, color='skyblue')
    plt.xlim(0, 150)

    bincenters = 0.5 * (bins[:-1] + bins[1:])
    s = min(bincenters)
```

```
    d = 0.9 * s

    popt, _ = curve_fit(lambda x, a, b: sigd(x, a, b, d), bincenters, freq, bounds=([2, 0], [4, 200]))
    fit = sigd(bincenters, popt[0], popt[1], d)

    all_fits.append((bincenters, fit, color, label))

    plt.xlim(0, 100)
    plt.ylim(0, 0.10)
    plt.xlabel('B Values [$  ^2$]')
    plt.ylabel('Frequency')
    plt.title(f'SIGD Fit {label} Data')
    plt.plot(bincenters, fit, color)
    plt.show()

# Combined plot
for bincenters, fit, color, label in all_fits:
    plt.plot(bincenters, fit, color, label=label)

plt.legend(loc="upper right")
plt.xlabel('B Values [$  ^2$]')
plt.ylabel('Frequency')
plt.title('SIGD Fit : CTXM')
plt.xlim(0, 100)
plt.ylim(0, 0.10)
plt.show()
```

Script to calculate the C_α RMSD

```python
#!/usr/bin/env python3
# -*- coding: utf-8 -*-
"""
Created on Wed Jul 17 10:51:55 2024

@author: gargi
"""

#!/usr/bin/env python3
# -*- coding: utf-8 -*-
"""
Created on Thu Jul 11 12:05:00 2024

@author: user
"""

from Bio.PDB.PDBParser import PDBParser
from Bio.SVDSuperimposer import SVDSuperimposer
import numpy as np
import matplotlib as mpl
import matplotlib.pyplot as plt

# Define amino acids
AA = ["ALA", "CYS", "ASP", "GLU", "PHE", "GLY", "HIS", "ILE", "LYS", "LEU", "MET",
    "ASN", "PRO", "GLN", "ARG", "SER", "THR", "VAL", "TRP", "TYR"]

# Define PDBs
p = PDBParser(QUIET=True)
structure_files = ["", "", "", "", ""]
structures = [p.get_structure(f"ctxm_{i*10}c", file) for i, file in enumerate(
    structure_files, start=1)]

# Superposition with SVD
def align(model1, model2, atom_types=["CA"]):
    model1_coords = [a.coord for a in model1[0].get_atoms() if a.parent.resname in
    AA and a.name in atom_types]
    model2_coords = [a.coord for a in model2[0].get_atoms() if a.parent.resname in
    AA and a.name in atom_types]
    si = SVDSuperimposer()
```

```
    si.set(np.array(model1_coords), np.array(model2_coords))
    si.run()
    return si

# Calculate RMSDs and populate the matrix
num_structures = len(structures)
C_aRMSD = np.zeros((num_structures, num_structures))

rmsds = []
for i in range(num_structures):
    for j in range(i, num_structures):
        si = align(structures[i], structures[j])
        rmsd_value = si.get_rms()
        rmsds.append(rmsd_value)
        C_aRMSD[i, j] = rmsd_value
        C_aRMSD[j, i] = rmsd_value  # Symmetric matrix

formatted_rmsds = ['%.3f' % elem for elem in rmsds]
print(formatted_rmsds)

structures_labels = ["10 C ", "20 C ", "30 C ", "40 C ", "50 C "]

# Plotting
fig, ax = plt.subplots()
im = ax.imshow(C_aRMSD)

plt.rcParams["font.family"] = "arial"
mpl.rc('text.latex', preamble=r'\usepackage{amsmath}')

# Show all ticks and label them with the respective list entries
ax.set_xticks(np.arange(len(structures_labels)), labels=structures_labels, weight="bold")
ax.set_yticks(np.arange(len(structures_labels)), labels=structures_labels, weight="bold")

# Loop over data dimensions and create text annotations
for i in range(len(structures_labels)):
    for j in range(len(structures_labels)):
        ax.text(j, i, f'{C_aRMSD[i, j]:.3f}', ha="center", va="center", color="w", weight="bold")

```

```
ax.set_title(r'C$_{\mathbf{\alpha}}$ Pairwise RMSD : CTX-M-14', weight="bold")

cbar = plt.colorbar(im, ax=ax)
cbar.set_label(label='RMSD($\mathbf{\AA}$)', rotation=-90.0, va="bottom", weight='bold')
cbar.ax.set_yticklabels(np.arange(0.00, 0.09, 0.01), fontweight='bold')

fig.tight_layout()
plt.rcParams['figure.dpi'] = 800
plt.rcParams['savefig.dpi'] = 800
plt.show()
```

Mathematica 10.4 code for the numerical calculations

```
Notebook[{
Cell[BoxData[
 RowBox[{
  RowBox[{"(*",
   RowBox[{"Spatial", " ", "domain", " ", "in", " ", "micrometers"}], "*)"}],
  "\[IndentingNewLine]",
  RowBox[{
   RowBox[{
    RowBox[{"size", "=", "50"}], ";"}], "\[IndentingNewLine]",
   RowBox[{
    RowBox[{
     RowBox[{"{",
      RowBox[{"xmin", ",", "xmax"}], "}"}], "=",
     RowBox[{"{",
      RowBox[{
       RowBox[{
        RowBox[{"-", "size"}], "/", "2"}], ",",
       RowBox[{"size", "/", "2"}]}]}], "}"}]}], ";"}], "\n",
   RowBox[{
    RowBox[{
     RowBox[{"{",
      RowBox[{"ymin", ",", "ymax"}], "}"}], "=",
     RowBox[{"{",
      RowBox[{
       RowBox[{
        RowBox[{"-", "size"}], "/", "2"}], ",",
       RowBox[{"size", "/", "2"}]}]}], "}"}]}], ";"}], "\[IndentingNewLine]",
   "\[IndentingNewLine]",
   RowBox[{"(*",
    RowBox[{"Thermal", " ", "diffusivities", " ", "in", " ",
     RowBox[{
      RowBox[{"\[Micro]m", "^", "2"}], "/", "ms"}]}]}], "*)"}],
   "\[IndentingNewLine]",
   RowBox[{
    RowBox[{"\[Alpha]XI", "=", "140"}], ";"}], "\[IndentingNewLine]",
   RowBox[{
    RowBox[{"\[Alpha]CTX", "=", "140"}], ";"}], "\[IndentingNewLine]",
   "\[IndentingNewLine]",
   RowBox[{"(*",
```

```
 RowBox[{"Exposure", " ", "times", " ", "in", " ", "ms"}], "*)"}], "\n",
RowBox[{
 RowBox[{"tExpXI", "=", "7"}], ";"}], "\[IndentingNewLine]",
RowBox[{
 RowBox[{"tExpCTX", "=", "5"}], ";"}], "\[IndentingNewLine]", "\n",
RowBox[{"(*",
 RowBox[{
  RowBox[{"Start", "/", "Stop"}], " ", "time", " ", "in", " ", "ms"}],
 "*)"}], "\n",
RowBox[{
 RowBox[{"tStart", "=",
  RowBox[{"-", "1"}]}], ";"}], "\[IndentingNewLine]",
RowBox[{
 RowBox[{"tStop", "=", "15"}], ";"}], "\[IndentingNewLine]",
"\[IndentingNewLine]",
RowBox[{"(*",
 RowBox[{
  RowBox[{
  "Beam", " ", "spatial", " ", "and", " ", "temporal", " ", "parameters"}],
   ",", " ",
  RowBox[{
  "conversion", " ", "from", " ", "FWHM", " ", "to", " ", "sigma"}]}],
 "*)"}], "\[IndentingNewLine]",
RowBox[{
 RowBox[{"sigmaX", "=",
  RowBox[{"30", "/",
   RowBox[{"(",
    RowBox[{"2",
     RowBox[{"Sqrt", "[",
      RowBox[{"2",
       RowBox[{"Log", "[", "2.", "]"}]}], "]"}]}], ")"}]}]}], ";"}], "\n",
RowBox[{
 RowBox[{"sigmaY", "=",
  RowBox[{"10", "/",
   RowBox[{"(",
    RowBox[{"2",
     RowBox[{"Sqrt", "[",
      RowBox[{"2",
       RowBox[{"Log", "[", "2.", "]"}]}], "]"}]}], ")"}]}]}], ";"}], "\n",
RowBox[{
 RowBox[{"aperture", "=", "10"}], ";"}], "\[IndentingNewLine]",
```

```
"\[IndentingNewLine]",
RowBox[{"(*",
 RowBox[{
 "Max", " ", "temperature", " ", "increase", " ", "at", " ", "center"}],
 "*)"}], "\n",
RowBox[{
 RowBox[{"deltaTempXI", "=", "20.12"}], ";"}], "\[IndentingNewLine]",
RowBox[{
 RowBox[{"deltaTempCTX", "=", "11.24"}], ";"}], "\[IndentingNewLine]",
"\[IndentingNewLine]",
RowBox[{"(*",
 RowBox[{
 "Smooth", " ", "rectangular", " ", "window", " ", "using", " ", "tanh",
  RowBox[{"(", ")"}]}], " ", "*)"}], "\[IndentingNewLine]",
RowBox[{
 RowBox[{
  RowBox[{"SmoothWindow", "[",
   RowBox[{"x_", ",", "w_", ",", "s_"}], "]"}], ":=",
  RowBox[{
   RowBox[{
    RowBox[{"Tanh", "[",
     RowBox[{
      RowBox[{"(",
       RowBox[{"x", "+",
        RowBox[{"w", "/", "2"}]}]}], ")"}], "/", "s"}], "]"}], "/", "2"}],
   "-",
   RowBox[{
    RowBox[{"Tanh", "[",
     RowBox[{
      RowBox[{"(",
       RowBox[{"x", "-",
        RowBox[{"w", "/", "2"}]}]}], ")"}], "/", "s"}], "]"}], "/",
    "2"}]}]}], ";"}], "\[IndentingNewLine]",
RowBox[{"(*",
 RowBox[{
  RowBox[{"Sharp", " ", "rectangular", " ", "window"}], ",", " ",
  RowBox[{"not", " ", "used"}]}]}], " ", "*)"}], "\n",
RowBox[{
 RowBox[{
  RowBox[{"SharpWindow", "[",
   RowBox[{"x_", ",", "w_", ",", "s_"}], "]"}], ":=",
```

```
    RowBox[{"DirichletWindow", "[",
     RowBox[{"x", "/", "w"}], "]"}]}], ";"}], " ", "\[IndentingNewLine]",
  "\[IndentingNewLine]",
  RowBox[{"(*",
   RowBox[{"Time", "-",
    RowBox[{"and", " ", "space"}], "-",
    RowBox[{"dependent", " ", "beam"}]}], "*)"}], "\[IndentingNewLine]",
  RowBox[{
   RowBox[{
    RowBox[{"beamRaw", "[",
     RowBox[{"x_", ",", "y_"}], "]"}], ":=",
    RowBox[{
     RowBox[{"Exp", "[",
      RowBox[{
       RowBox[{"-",
        RowBox[{"x", "^", "2"}]}], "/",
       RowBox[{"(",
        RowBox[{"2", " ",
         RowBox[{"sigmaX", "^", "2"}]}], ")"}]}], "]"}], "*",
     RowBox[{"Exp", "[",
      RowBox[{
       RowBox[{"-",
        RowBox[{"y", "^", "2"}]}], "/",
       RowBox[{"(",
        RowBox[{"2", " ",
         RowBox[{"sigmaY", "^", "2"}]}], ")"}]}], "]"}]}]}], ";"}],
  "\[IndentingNewLine]",
  RowBox[{
   RowBox[{
    RowBox[{"beamAperture", "[",
     RowBox[{"x_", ",", "y_"}], "]"}], ":=",
    RowBox[{
     RowBox[{"beamRaw", "[",
      RowBox[{"x", ",", "y"}], "]"}], "*",
     RowBox[{"SmoothWindow", "[",
      RowBox[{
       RowBox[{"Sqrt", "[",
        RowBox[{
         RowBox[{"x", "^", "2"}], "+",
         RowBox[{"y", "^", "2"}]}]}], "]"}], ",", "aperture", ",", "0.2"}],
      "]"}]}]}], ";"}], "\[IndentingNewLine]",
```

```
RowBox[{
 RowBox[{
  RowBox[{"temporalProfile", "[",
   RowBox[{"t_", ",", "tExp_"}], "]"}], ":=",
  RowBox[{"SmoothWindow", "[",
   RowBox[{
    RowBox[{"t", "-",
     RowBox[{"tExp", "/", "2"}]}]}], ",", "tExp", ",", "0.05"}], "]"}]}],
 ";"}], "\[IndentingNewLine]", "\[IndentingNewLine]",
RowBox[{"(*",
 RowBox[{"Beam", " ", "shape", " ", "visualizations"}], "*)"}],
"\[IndentingNewLine]",
RowBox[{
 RowBox[{"P1", "=",
  RowBox[{"Plot", "[",
   RowBox[{
    RowBox[{"{",
     RowBox[{
      RowBox[{"beamRaw", "[",
       RowBox[{"x", ",", "0"}], "]"}], ",",
      RowBox[{"beamAperture", "[",
       RowBox[{"x", ",", "0"}], "]"}]}]}], "}"}], ",",
    RowBox[{"{",
     RowBox[{"x", ",",
      RowBox[{"-", "15"}], ",", "15"}], "}"}], ",", "\[IndentingNewLine]",
    RowBox[{
    "PlotLabel", "\[Rule]",
     "\"\<Normalized beam profile along x at y=0\>\""}], ",",
    RowBox[{"AxesLabel", "\[Rule]",
     RowBox[{"{",
      RowBox[{"\"\<x\>\"", ",", "\"\<Beam(x,0)\>\""}], "}"}]}], ",",
    "\[IndentingNewLine]",
    RowBox[{"PlotRange", "\[Rule]", "All"}], ",",
    RowBox[{"PlotStyle", "\[Rule]",
     RowBox[{"{",
      RowBox[{"Dashed", ",", "Thick"}], "}"}]}], ",",
    "\[IndentingNewLine]",
    RowBox[{"PlotLegends", "\[Rule]",
     RowBox[{"Placed", "[",
      RowBox[{
       RowBox[{"{",
```

```
          RowBox[{"\"\<Raw beam\>\"", ",", "\"\<With aperture\>\""}], "}"}],
         ",",
         RowBox[{"Scaled", "[",
          RowBox[{"{",
           RowBox[{"0.85", ",", "0.33"}], "}"}], "]"}]}], "]"}]}], ",",
      "\[IndentingNewLine]",
      RowBox[{"ImageSize", "\[Rule]", "400"}]}], "\[IndentingNewLine]",
     "]"}]}], ";"}], "\[IndentingNewLine]",
 RowBox[{
  RowBox[{"P2", "=",
   RowBox[{"Plot", "[",
    RowBox[{
      RowBox[{"{",
       RowBox[{
        RowBox[{"beamRaw", "[",
         RowBox[{"0", ",", "y"}], "]"}], ",",
        RowBox[{"beamAperture", "[",
         RowBox[{"0", ",", "y"}], "]"}]}], "}"}], ",",
      RowBox[{"{",
       RowBox[{"y", ",",
        RowBox[{"-", "15"}], ",", "15"}], "}"}], ",", "\[IndentingNewLine]",
      RowBox[{
      "PlotLabel", "\[Rule]",
       "\"\<Normalized beam profile along y at x=0\>\""}], ",",
      RowBox[{"AxesLabel", "\[Rule]",
       RowBox[{"{",
        RowBox[{"\"\<y\>\"", ",", "\"\<Beam(0,y)\>\""}], "}"}]}], ",",
      "\[IndentingNewLine]",
      RowBox[{"PlotRange", "\[Rule]", "All"}], ",",
      RowBox[{"PlotStyle", "\[Rule]",
       RowBox[{"{",
        RowBox[{"Dashed", ",", "Thick"}], "}"}]}], ",",
      "\[IndentingNewLine]",
      RowBox[{"PlotLegends", "\[Rule]",
       RowBox[{"Placed", "[",
        RowBox[{
         RowBox[{"{",
          RowBox[{"\"\<Raw beam\>\"", ",", "\"\<With aperture\>\""}], "}"}],
         ",",
         RowBox[{"Scaled", "[",
          RowBox[{"{",
```

```
         RowBox[{"0.85", ",", "0.66"}], "}"}], "]"}]}], "]"}]}], ",",
    "\[IndentingNewLine]",
    RowBox[{"ImageSize", "\[Rule]", "400"}]}], "\[IndentingNewLine]",
   "]"}]}], ";"}], "\n",
RowBox[{
 RowBox[{"P3", "=",
  RowBox[{"Plot", "[",
   RowBox[{
    RowBox[{"{",
     RowBox[{
      RowBox[{"temporalProfile", "[",
       RowBox[{"t", ",", "tExpXI"}], "]"}], ",",
      RowBox[{"temporalProfile", "[",
       RowBox[{"t", ",", "tExpCTX"}], "]"}]}], "}"}], ",",
    RowBox[{"{",
     RowBox[{"t", ",",
      RowBox[{"-", "1"}], ",", "11"}], "}"}], ",", "\[IndentingNewLine]",
    RowBox[{
    "PlotLabel", "\[Rule]",
     "\"\<Normalized temporal beam profile at center\>\""}], ",",
    "\[IndentingNewLine]",
    RowBox[{"AxesLabel", "\[Rule]",
     RowBox[{"{",
      RowBox[{"\"\<t\>\"", ",", "\"\<Beam(0,0,t)\>\""}], "}"}]}], ",",
    "\[IndentingNewLine]",
    RowBox[{"AxesOrigin", "\[Rule]",
     RowBox[{"{",
      RowBox[{"tStart", ",", "0"}], "}"}]}], ",", "\[IndentingNewLine]",
    RowBox[{"PlotLegends", "\[Rule]",
     RowBox[{"Placed", "[",
      RowBox[{
       RowBox[{"{",
        RowBox[{"\"\<XI: 7 ms\>\"", ",", "\"\<CTX: 5 ms\>\""}], "}"}], ",",
       RowBox[{"Scaled", "[",
        RowBox[{"{",
         RowBox[{"0.85", ",", "0.66"}], "}"}], "]"}]}], "]"}]}], ",",
    "\[IndentingNewLine]",
    RowBox[{"ImageSize", "\[Rule]", "400"}]}], "\[IndentingNewLine]",
   "]"}]}], ";"}], "\[IndentingNewLine]", "\[IndentingNewLine]",
RowBox[{"(*",
 RowBox[{"Pure", " ", "source", " ", "accumulation", " ",
```

```
    RowBox[{"(",
     RowBox[{"no", " ", "diffusion"}], ")"}]}], "*)"}],
  "\[IndentingNewLine]",
  RowBox[{
   RowBox[{"solutionXIstat", "=",
    RowBox[{"NDSolveValue", "[",
     RowBox[{
      RowBox[{"{",
       RowBox[{
        RowBox[{
         RowBox[{"D", "[",
          RowBox[{
           RowBox[{"u", "[",
            RowBox[{"x", ",", "y", ",", "t"}], "]"}], ",", "t"}], "]"}],
         "\[Equal]",
         RowBox[{
          RowBox[{"beamAperture", "[",
           RowBox[{"x", ",", "y"}], "]"}], "*",
          RowBox[{"temporalProfile", "[",
           RowBox[{"t", ",", "tExpXI"}], "]"}],
          RowBox[{"deltaTempXI", "/", "tExpXI"}]}]}]}], ",",
        RowBox[{
         RowBox[{"u", "[",
          RowBox[{"x", ",", "y", ",", "tStart"}], "]"}], "\[Equal]", "0"}]}],
        "}"}], ",", "u", ",",
      RowBox[{"{",
       RowBox[{"x", ",", "xmin", ",", "xmax"}], "}"}], ",",
      RowBox[{"{",
       RowBox[{"y", ",", "ymin", ",", "ymax"}], "}"}], ",",
      RowBox[{"{",
       RowBox[{"t", ",", "tStart", ",", "tStop"}], "}"}]}], "]"}]}], ";"}],
  "\[IndentingNewLine]",
  RowBox[{
   RowBox[{"solutionCTXstat", "=",
    RowBox[{"NDSolveValue", "[",
     RowBox[{
      RowBox[{"{",
       RowBox[{
        RowBox[{
         RowBox[{"D", "[",
          RowBox[{
```

```
              RowBox[{"u", "[",
               RowBox[{"x", ",", "y", ",", "t"}], "]"}], ",", "t"}], "]"}],
            "\[Equal]",
            RowBox[{
             RowBox[{"beamAperture", "[",
              RowBox[{"x", ",", "y"}], "]"}], "*",
             RowBox[{"temporalProfile", "[",
              RowBox[{"t", ",", "tExpCTX"}], "]"}],
             RowBox[{"deltaTempCTX", "/", "tExpCTX"}]}]}]}], ",",
           RowBox[{
            RowBox[{"u", "[",
             RowBox[{"x", ",", "y", ",", "tStart"}], "]"}], "\[Equal]", "0"}]}]},
           "}"}], ",", "u", ",",
         RowBox[{"{",
          RowBox[{"x", ",", "xmin", ",", "xmax"}], "}"}], ",",
         RowBox[{"{",
          RowBox[{"y", ",", "ymin", ",", "ymax"}], "}"}], ",",
         RowBox[{"{",
          RowBox[{"t", ",", "tStart", ",", "tStop"}], "}"}]}]}], "]"}]}]}], ";"}],
  "\[IndentingNewLine]", "\[IndentingNewLine]",
  RowBox[{"(*",
   RowBox[{"Heat", " ", "diffusion", " ", "with", " ", "source"}], "*)"}],
  "\[IndentingNewLine]",
  RowBox[{
   RowBox[{"solutionXIdyn", "=",
    RowBox[{"NDSolveValue", "[",
     RowBox[{
      RowBox[{"{",
       RowBox[{
        RowBox[{
         RowBox[{"D", "[",
          RowBox[{
           RowBox[{"u", "[",
            RowBox[{"x", ",", "y", ",", "t"}], "]"}], ",", "t"}], "]"}],
         "\[Equal]",
         RowBox[{
          RowBox[{"\[Alpha]XI", "*",
           RowBox[{"Laplacian", "[",
            RowBox[{
             RowBox[{"u", "[",
              RowBox[{"x", ",", "y", ",", "t"}], "]"}], ",",
```

```
       RowBox[{"{",
        RowBox[{"x", ",", "y"}], "}"}]}], "]"}]}], "+",
    RowBox[{
     RowBox[{"beamAperture", "[",
      RowBox[{"x", ",", "y"}], "]"}], "*",
     RowBox[{"temporalProfile", "[",
      RowBox[{"t", ",", "tExpXI"}], "]"}],
     RowBox[{"deltaTempXI", "/", "tExpXI"}]}]}]}]}], ",",
  RowBox[{
   RowBox[{"u", "[",
    RowBox[{"x", ",", "y", ",", "tStart"}], "]"}], "\[Equal]", "0"}],
  ",", " ",
  RowBox[{"DirichletCondition", "[",
   RowBox[{
    RowBox[{
     RowBox[{"u", "[",
      RowBox[{"x", ",", "y", ",", "t"}], "]"}], "\[Equal]", "0"}], ",",
    RowBox[{
     RowBox[{"Abs", "[",
      RowBox[{
       RowBox[{"x", "^", "2"}], "+",
       RowBox[{"y", "^", "2"}], "-",
       RowBox[{
        RowBox[{"(",
         RowBox[{"size", "/", "2"}], ")"}], "^", "2"}]}]}], "]"}], "<",
     "0.1"}]}]}], "]"}]}]}], "}"}], ",", "u", ",",
RowBox[{"{",
 RowBox[{"x", ",", "xmin", ",", "xmax"}], "}"}], ",",
RowBox[{"{",
 RowBox[{"y", ",", "ymin", ",", "ymax"}], "}"}], ",",
RowBox[{"{",
 RowBox[{"t", ",", "tStart", ",", "tStop"}], "}"}], ",",
RowBox[{"Method", "\[Rule]",
 RowBox[{"{",
  RowBox[{"\"\<MethodOfLines\>\"", ",",
   RowBox[{"\"\<SpatialDiscretization\>\"", "\[Rule]",
    RowBox[{"{",
     RowBox[{"\"\<FiniteElement\>\"", ",",
      RowBox[{"\"\<MeshOptions\>\"", "\[Rule]",
       RowBox[{"{",
        RowBox[{"\"\<MaxCellMeasure\>\"", "\[Rule]", "0.5"}],
```

```
              "}"}]}]}], "}"}]}]}], "}"}]}]}], "]"}]}], ";"}],
"\[IndentingNewLine]",
RowBox[{
 RowBox[{"solutionCTXdyn", "=",
  RowBox[{"NDSolveValue", "[",
   RowBox[{
    RowBox[{"{",
     RowBox[{
      RowBox[{
       RowBox[{"D", "[",
        RowBox[{
         RowBox[{"u", "[",
          RowBox[{"x", ",", "y", ",", "t"}], "]"}], ",", "t"}], "]"}],
       "\[Equal]",
       RowBox[{
        RowBox[{"\[Alpha]CTX", "*",
         RowBox[{"Laplacian", "[",
          RowBox[{
           RowBox[{"u", "[",
            RowBox[{"x", ",", "y", ",", "t"}], "]"}], ",",
           RowBox[{"{",
            RowBox[{"x", ",", "y"}], "}"}]}], "]"}]}], "+",
        RowBox[{
         RowBox[{"beamAperture", "[",
          RowBox[{"x", ",", "y"}], "]"}], "*",
         RowBox[{"temporalProfile", "[",
          RowBox[{"t", ",", "tExpCTX"}], "]"}],
         RowBox[{"deltaTempCTX", "/", "tExpCTX"}]}]}]}], ",",
      RowBox[{
       RowBox[{"u", "[",
        RowBox[{"x", ",", "y", ",", "tStart"}], "]"}], "\[Equal]", "0"}],
      ",",
      RowBox[{"DirichletCondition", "[",
       RowBox[{
        RowBox[{
         RowBox[{"u", "[",
          RowBox[{"x", ",", "y", ",", "t"}], "]"}], "\[Equal]", "0"}], ",",
        RowBox[{
         RowBox[{"Abs", "[",
          RowBox[{
           RowBox[{"x", "^", "2"}], "+",
```

```
           RowBox[{"y", "^", "2"}], "-",
           RowBox[{
            RowBox[{"(",
             RowBox[{"size", "/", "2"}], ")"}], "^", "2"}]}], "]"}], "<",
         "0.1"}]}], "]"}]}], "}"}], ",", "u", ",",
    RowBox[{"{",
     RowBox[{"x", ",", "xmin", ",", "xmax"}], "}"}], ",",
    RowBox[{"{",
     RowBox[{"y", ",", "ymin", ",", "ymax"}], "}"}], ",",
    RowBox[{"{",
     RowBox[{"t", ",", "tStart", ",", "tStop"}], "}"}], ",",
    RowBox[{"Method", "\[Rule]",
     RowBox[{"{",
      RowBox[{"\"\<MethodOfLines\>\"", ",",
       RowBox[{"\"\<SpatialDiscretization\>\"", "\[Rule]",
        RowBox[{"{",
         RowBox[{"\"\<FiniteElement\>\"", ",",
          RowBox[{"\"\<MeshOptions\>\"", "\[Rule]",
           RowBox[{"{",
            RowBox[{"\"\<MaxCellMeasure\>\"", "\[Rule]", "0.5"}],
            "}"}]}]}]}], "}"}]}]}]}], "}"}]}]}]}], "]"}]}]}], ";"}],
"\[IndentingNewLine]", "\[IndentingNewLine]",
RowBox[{"(*", "Export", "*)"}], "\[IndentingNewLine]",
RowBox[{
 RowBox[{"Export", "[",
  RowBox[{
   RowBox[{"FileNameJoin", "[",
    RowBox[{"{",
     RowBox[{
      RowBox[{"NotebookDirectory", "[", "]"}], ",",
      "\"\<beam profile X.txt\>\""}], " ", "}"}], "]"}], ",",
   RowBox[{"N", "@",
    RowBox[{"Round", "[",
     RowBox[{
      RowBox[{"Table", "[",
       RowBox[{
        RowBox[{"{",
         RowBox[{"x", ",",
          RowBox[{"beamRaw", "[",
           RowBox[{"x", ",", "0"}], "]"}], ",",
          RowBox[{"beamAperture", "[",
```

```
           RowBox[{"x", ",", "0"}], "]"}]}], "}"}], ",",
        RowBox[{"{",
         RowBox[{"x", ",",
          RowBox[{"-", "15"}], ",", "15", ",", "0.01"}], "}"}]}], "]"}],
      ",",
      RowBox[{"1", "/", "1000"}]}], "]"}]}]}], "]"}], ";"}],
"\[IndentingNewLine]",
RowBox[{
 RowBox[{"Export", "[",
  RowBox[{
   RowBox[{"FileNameJoin", "[",
    RowBox[{"{",
     RowBox[{
      RowBox[{"NotebookDirectory", "[", "]"}], ",",
      "\"\<beam profile Y.txt\>\""}], " ", "}"}], "]"}], ",",
   RowBox[{"N", "@",
    RowBox[{"Round", "[",
     RowBox[{
      RowBox[{"Table", "[",
       RowBox[{
        RowBox[{"{",
         RowBox[{"y", ",",
          RowBox[{"beamRaw", "[",
           RowBox[{"0", ",", "y"}], "]"}], ",",
          RowBox[{"beamAperture", "[",
           RowBox[{"0", ",", "y"}], "]"}]}], "}"}], ",",
        RowBox[{"{",
         RowBox[{"y", ",",
          RowBox[{"-", "15"}], ",", "15", ",", "0.01"}], "}"}]}], "]"}],
      ",",
      RowBox[{"1", "/", "1000"}]}], "]"}]}]}], "]"}], ";"}],
"\[IndentingNewLine]",
RowBox[{
 RowBox[{"Export", "[",
  RowBox[{
   RowBox[{"FileNameJoin", "[",
    RowBox[{"{",
     RowBox[{
      RowBox[{"NotebookDirectory", "[", "]"}], ",",
      "\"\<beam profile t.txt\>\""}], " ", "}"}], "]"}], ",",
   RowBox[{"N", "@",
```

```
        RowBox[{"Round", "[",
         RowBox[{
          RowBox[{"Table", "[",
           RowBox[{
            RowBox[{"{",
             RowBox[{"t", ",",
              RowBox[{"temporalProfile", "[",
               RowBox[{"t", ",", "tExpXI"}], "]"}], ",",
              RowBox[{"temporalProfile", "[",
               RowBox[{"t", ",", "tExpCTX"}], "]"}]}], "}"}], ",",
            RowBox[{"{",
             RowBox[{"t", ",",
              RowBox[{"-", "1"}], ",", "11", ",", "0.01"}], "}"}]}], "]"}],
          ",",
          RowBox[{"1", "/", "1000"}]}]}], "]"}]}]}], "]"}], ";"}],
    "\[IndentingNewLine]",
    RowBox[{
     RowBox[{"Export", "[",
      RowBox[{
       RowBox[{"FileNameJoin", "[",
        RowBox[{"{",
         RowBox[{
          RowBox[{"NotebookDirectory", "[", "]"}], ",",
          "\"\<heatexchange_ms_K_alpha140.txt\>\""}], " ", "}"}], "]"}], ",",
       RowBox[{"N", "@",
        RowBox[{"Round", "[",
         RowBox[{
          RowBox[{"Table", "[",
           RowBox[{
            RowBox[{"{",
             RowBox[{"t", ",",
              RowBox[{"solutionXIstat", "[",
               RowBox[{"0", ",", "0", ",", "t"}], "]"}], ",",
              RowBox[{"solutionXIdyn", "[",
               RowBox[{"0", ",", "0", ",", "t"}], "]"}], ",",
              RowBox[{"solutionCTXstat", "[",
               RowBox[{"0", ",", "0", ",", "t"}], "]"}], ",",
              RowBox[{"solutionCTXdyn", "[",
               RowBox[{"0", ",", "0", ",", "t"}], "]"}]}], "}"}], ",",
            RowBox[{"{",
             RowBox[{"t", ",", "tStart", ",", "tStop", ",", "0.01"}], "}"}]}],
```

```
          "]"}], ",",
         RowBox[{"1", "/", "1000"}]}], "]"}]}]}], "]"}], ";"}]}]}]], "Input",
 CellChangeTimes->{{3.9569200916785126`*^9, 3.956920101102765*^9}, {
   3.9569201661364965`*^9, 3.9569201929664497`*^9}, {3.9569202292615585`*^9,
   3.956920258230592*^9}, 3.9569842602131667`*^9, {3.9569843921419907`*^9,
   3.9569844036068506`*^9}, {3.9569844549562674`*^9,
   3.9569844611040573`*^9}, {3.956984613293849*^9, 3.956984615722562*^9}, {
   3.9569855463363967`*^9, 3.9569855480858684`*^9}, {3.9569988612522793`*^9,
   3.9569988621214633`*^9}, {3.9569989594437413`*^9, 3.956998962122645*^9}, {
   3.9569991128612237`*^9, 3.9569991143703966`*^9}, {3.956999292864998*^9,
   3.9569992943838105`*^9}, {3.9569993962577596`*^9, 3.956999397087318*^9}, {
   3.957260028523471*^9, 3.9572600350511436`*^9}, {3.957260879319916*^9,
   3.9572608819598885`*^9}, {3.957261430169819*^9, 3.957261432474338*^9}, {
   3.9572614911704416`*^9, 3.9572614935943956`*^9}, {3.9572616347388515`*^9,
   3.9572616352746925`*^9}, {3.957339302494481*^9, 3.9573393097517886`*^9}, {
   3.957340101787078*^9, 3.9573401019569798`*^9}, {3.9573406842538385`*^9,
   3.957340684727606*^9}, {3.9573432010843644`*^9, 3.957343207101162*^9}, {
   3.957854602622549*^9, 3.957854652719934*^9}, {3.9578546913373027`*^9,
   3.9578547317128434`*^9}}],

Cell[BoxData[
 RowBox[{
  RowBox[{"(*",
   RowBox[{"Data", " ", "plot"}], "*)"}], "\[IndentingNewLine]",
  RowBox[{
   RowBox[{
    RowBox[{"Row", "@",
     RowBox[{"{",
      RowBox[{"P1", ",", "P2", ",", "P3"}], "}"}]}]}], ";"}],
   "\[IndentingNewLine]",
   RowBox[{
    RowBox[{"C1", "=",
     RowBox[{"RGBColor", "[",
      RowBox[{"0.368417", ",", "0.506779", ",", "0.709798"}], "]"}]}]}], ";",
    " ",
    RowBox[{"C2", "=",
     RowBox[{"RGBColor", "[",
      RowBox[{"0.880722", ",", "0.611041", ",", "0.142051"}], "]"}]}]}], ";"}],
   "\[IndentingNewLine]",
   RowBox[{
    RowBox[{"Plot", "[",
```

```
    RowBox[{
     RowBox[{"{",
      RowBox[{
       RowBox[{"solutionXIstat", "[",
        RowBox[{"0", ",", "0", ",", "t"}], "]"}], ",",
       RowBox[{"10",
        RowBox[{"solutionXIdyn", "[",
         RowBox[{"0", ",", "0", ",", "t"}], "]"}]}], ",",
       RowBox[{"solutionCTXstat", "[",
        RowBox[{"0", ",", "0", ",", "t"}], "]"}], ",",
       RowBox[{"10",
        RowBox[{"solutionCTXdyn", "[",
         RowBox[{"0", ",", "0", ",", "t"}], "]"}]}]}], "}"}], ",",
     RowBox[{"{",
      RowBox[{"t", ",", "tStart", ",", "15"}], "}"}], ",",
     "\[IndentingNewLine]",
     RowBox[{"PlotLegends", "\[Rule]",
      RowBox[{"Placed", "[",
       RowBox[{
        RowBox[{"{",
         RowBox[{
         "\"\<XI no diffusion\>\"", ",", "\"\<XI with diffusion (10x)\>\"",
          ",", "\"\<CTX no diffusion\>\"", ",",
          "\"\<CTX with diffusion (10x)\>\""}], "}"}], ",",
        RowBox[{"Scaled", "[",
         RowBox[{"{",
          RowBox[{"0.8", ",", "0.75"}], "}"}], "]"}]}], "]"}]}], ",",
     "\[IndentingNewLine]",
     RowBox[{
     "PlotLabel", "\[Rule]",
      "\"\<Temperature rise at center over time\>\""}], ",",
     "\[IndentingNewLine]",
     RowBox[{"AxesOrigin", "\[Rule]",
      RowBox[{"{",
       RowBox[{"tStart", ",", "0"}], "}"}]}], ",",
     RowBox[{"AxesLabel", "\[Rule]",
      RowBox[{"{",
       RowBox[{"\"\<Time (ms)\>\"", ",", "\"\<Temperature (K)\>\""}],
       "}"}]}], ",",
     RowBox[{"PlotRange", "\[Rule]", "All"}], ",",
     RowBox[{"PlotStyle", "\[Rule]",
```

```
       RowBox[{"{",
        RowBox[{
         RowBox[{"{",
          RowBox[{"C1", ",", "Dashed"}], "}"}], ",",
         RowBox[{"{",
          RowBox[{"C1", ",", "Thick"}], "}"}], ",",
         RowBox[{"{",
          RowBox[{"C2", ",", "Dashed"}], "}"}], ",",
         RowBox[{"{",
          RowBox[{"C2", ",", "Thick"}], "}"}]}], "}"}]}], ",",
      RowBox[{"ImageSize", "\[Rule]", "600"}]}], "]"}], ";"}]}]}]], "Input",
 CellChangeTimes->{{3.9569148042295437`*^9, 3.9569148046192536`*^9}, {
  3.9569845415368876`*^9, 3.956984572925686*^9}, {3.9569856727816863`*^9,
  3.9569856849656987`*^9}, {3.9569860328435955`*^9, 3.9569860628613167`*^9}, {
  3.956986092901287*^9, 3.956986162453848*^9}, {3.956986329863558*^9,
  3.9569863668966184`*^9}, {3.956986404838026*^9, 3.956986423573355*^9}, {
  3.9569865131787095`*^9, 3.9569865194444857`*^9}, {3.956986578329771*^9,
  3.956986642132743*^9}, {3.957000089025634*^9, 3.957000106851861*^9}, {
  3.957260389605113*^9, 3.9572604009759307`*^9}, {3.9572615244828415`*^9,
  3.9572615615794544`*^9}, {3.9572617765312705`*^9, 3.9572617849713016`*^9}, {
  3.9573432391329856`*^9, 3.9573432572593904`*^9}, {3.957854643271599*^9,
  3.9578546436881337`*^9}, {3.957854812889577*^9, 3.9578548194174414`*^9}}]
},
WindowSize->{1831, 920},
WindowMargins->{{36, Automatic}, {Automatic, 31}},
PrintingCopies->1,
PrintingPageRange->{32000, 32000},
PrintingOptions->{"Magnification"->1.,
"PaperOrientation"->"Portrait",
"PaperSize"->{595.02, 841.98}},
FrontEndVersion->"10.4 for Microsoft Windows (64-bit) (February 25, 2016)",
StyleDefinitions->"Default.nb"
]
```

Supplementary References

[1] Knight, A. L., Widjaja, V. & Lisi, G. P. Temperature as a modulator of allosteric motions and crosstalk in mesophilic and thermophilic enzymes. *Frontiers in Molecular Biosciences* **10**, 1281062 (2023).

[2] Knapp, B. D. & Huang, K. C. The effects of temperature on cellular physiology. *Annual Review of Biophysics* **51**, 499–526 (2022).

[3] Winter, S. D. *et al.* Chemical mapping exposes the importance of active site interactions in governing the temperature dependence of enzyme turnover. *ACS catalysis* **11**, 14854–14863 (2021).

[4] Garman, E. F. Radiation damage in macromolecular crystallography: what is it and why should we care? *Acta Crystallographica Section D: Biological Crystallography* **66**, 339–351 (2010).

[5] Halle, B. Biomolecular cryocrystallography: structural changes during flash-cooling. *Proceedings of the National Academy of Sciences* **101**, 4793–4798 (2004).

[6] Fraser, J. S. *et al.* Hidden alternative structures of proline isomerase essential for catalysis. *Nature* **462**, 669–673 (2009).

[7] Fraser, J. S. *et al.* Accessing protein conformational ensembles using room-temperature x-ray crystallography. *Proceedings of the National Academy of Sciences* **108**, 16247–16252 (2011).

[8] Keedy, D. A. *et al.* Crystal cryocooling distorts conformational heterogeneity in a model michaelis complex of dhfr. *Structure* **22**, 899–910 (2014).

[9] Keedy, D. A. *et al.* Mapping the conformational landscape of a dynamic enzyme by multitemperature and xfel crystallography. *Elife* **4**, e07574 (2015).

[10] Mehlman, T. S. *et al.* Room-temperature crystallography reveals altered binding of small-molecule fragments to ptp1b. *Elife* **12**, e84632 (2023).

[11] Du, S. *et al.* Refinement of multiconformer ensemble models from multi-temperature x-ray diffraction data. *Methods in enzymology* **688**, 223–254 (2023).

[12] Bradford, S. Y. *et al.* Temperature artifacts in protein structures bias ligand-binding predictions. *Chemical Science* **12**, 11275–11293 (2021).

[13] Gerlits, O. *et al.* Room temperature neutron crystallography of drug resistant hiv-1 protease uncovers limitations of x-ray structural analysis at 100 k. *Journal of medicinal chemistry* **60**, 2018–2025 (2017).

[14] Strauch, M. & Heyd, F. Temperature does matter—an additional dimension in kinase inhibitor development. *The FEBS Journal* **288**, 3148–3153 (2021).

[15] Schiebel, J. *et al.* Intriguing role of water in protein-ligand binding studied by neutron crystallography on trypsin complexes. *Nature communications* **9**, 3559 (2018).

[16] Stachowski, T. R., Vanarotti, M., Seetharaman, J., Lopez, K. & Fischer, M. Water networks repopulate protein–ligand interfaces with temperature. *Angewandte Chemie* **134**, e202112919 (2022).

[17] Eisenmesser, E. Z., Bosco, D. A., Akke, M. & Kern, D. Enzyme dynamics during catalysis. *Science* **295**, 1520–1523 (2002).

[18] Henzler-Wildman, K. & Kern, D. Dynamic personalities of proteins. *Nature* **450**, 964–972 (2007).

[19] Freiburger, L. A. *et al.* Competing allosteric mechanisms modulate substrate binding in a dimeric enzyme. *Nature structural & molecular biology* **18**, 288–294 (2011).

[20] Motlagh, H. N., Wrabl, J. O., Li, J. & Hilser, V. J. The ensemble nature of allostery. *Nature* **508**, 331–339 (2014).

[21] Arcus, V. L., Van Der Kamp, M. W., Pudney, C. R. & Mulholland, A. J. Enzyme evolution and the temperature dependence of enzyme catalysis. *Current Opinion in Structural Biology* **65**, 96–101 (2020).

[22] Ebrahim, A. *et al.* The temperature-dependent conformational ensemble of sars-cov-2 main protease (mpro). *IUCrJ* **9**, 682–694 (2022).

[23] Jumper, J. *et al.* Highly accurate protein structure prediction with alphafold. *nature* **596**, 583–589 (2021).

[24] Baek, M. *et al.* Accurate prediction of protein structures and interactions using a three-track neural network. *Science* **373**, 871–876 (2021).

[25] Lin, Z. *et al.* Evolutionary-scale prediction of atomic-level protein structure with a language model. *Science* **379**, 1123–1130 (2023).

[26] Ahdritz, G. *et al.* Openfold: Retraining alphafold2 yields new insights into its learning mechanisms and capacity for generalization. *Nature Methods* 1–11 (2024).

[27] Terwilliger, T. C. *et al.* Alphafold predictions are valuable hypotheses and accelerate but do not replace experimental structure determination. *Nature Methods* **21**, 110–116 (2024).

[28] Pearson, A. R. & Mehrabi, P. Serial synchrotron crystallography for time-resolved structural biology. *Current Opinion in Structural Biology* **65**, 168–174 (2020).

[29] Chapman, H. N. X-ray free-electron lasers for the structure and dynamics of macromolecules. *Annual review of biochemistry* **88**, 35–58 (2019).

[30] Schmidt, M., Graber, T., Henning, R. & Srajer, V. Five-dimensional crystallography. *Acta Crystallographica Section A: Foundations of Crystallography* **66**, 198–206 (2010).

[31] Schmidt, M. *et al.* Protein energy landscapes determined by five-dimensional crystallography. *Acta Crystallographica Section D: Biological Crystallography* **69**, 2534–2542 (2013).

[32] Wolff, A. M. *et al.* Mapping protein dynamics at high spatial resolution with temperature-jump x-ray crystallography. *Nature chemistry* **15**, 1549–1558 (2023).

[33] Bar-Even, A. *et al.* The moderately efficient enzyme: evolutionary and physicochemical trends shaping enzyme parameters. *Biochemistry* **50**, 4402–4410 (2011).

[34] Perutz, M. The composition and swelling properties of haemoglobin crystals. *Transactions of the Faraday Society* **42**, B187–B195 (1946).

[35] Huxley, H. & Kendrew, J. Discontinuous lattice changes in haemoglobin crystals. *Acta Crystallographica* **6**, 76–80 (1953).

[36] Kiefersauer, R. *et al.* A novel free-mounting system for protein crystals: transformation and improvement of diffraction power by accurately controlled humidity

changes. *Journal of applied crystallography* **33**, 1223–1230 (2000).

[37] Park, H., Tran, T., Lee, J. H., Park, H. & Disney, M. D. Controlled dehydration improves the diffraction quality of two rna crystals. *BMC Structural Biology* **16**, 1–6 (2016).

[38] Lobley, C. M. *et al.* A generic protocol for protein crystal dehydration using the hc1b humidity controller. *Acta Crystallographica Section D: Structural Biology* **72**, 629–640 (2016).

[39] Bowler, M. W. *et al.* Automation and experience of controlled crystal dehydration: results from the european synchrotron hc1 collaboration. *Crystal Growth & Design* **15**, 1043–1054 (2015).

[40] Schulz, E. C. *et al.* The hit-and-return system enables efficient time-resolved serial synchrotron crystallography. *Nature methods* **15**, 901–904 (2018).

[41] de La Mora, E. *et al.* Radiation damage and dose limits in serial synchrotron crystallography at cryo-and room temperatures. *Proceedings of the National Academy of Sciences* **117**, 4142–4151 (2020).

[42] Mehrabi, P. *et al.* Serial femtosecond and serial synchrotron crystallography can yield data of equivalent quality: A systematic comparison. *Science advances* **7**, eabf1380 (2021).

[43] Mehrabi, P. *et al.* Liquid application method for time-resolved analyses by serial synchrotron crystallography. *Nature methods* **16**, 979–982 (2019).

[44] Monteiro, D. C., Amoah, E., Rogers, C. & Pearson, A. R. Using photocaging for fast time-resolved structural biology studies. *Acta Crystallographica Section D: Structural Biology* **77**, 1218–1232 (2021).

[45] Schulz, E. C., Yorke, B. A., Pearson, A. R. & Mehrabi, P. Best practices for time-resolved serial synchrotron crystallography. *Acta Crystallographica Section D: Structural Biology* **78**, 14–29 (2022).

[46] Ishii, Y., Galleni, M., Ma, L., Frère, J.-M. & Yamaguchi, K. Biochemical characterisation of the ctx-m-14 β-lactamase. *International journal of antimicrobial agents* **29**, 159–164 (2007).

[47] Bhosale, S. H., Rao, M. B. & Deshpande, V. V. Molecular and industrial aspects of glucose isomerase. *Microbiological reviews* **60**, 280–300 (1996).

[48] Nam, K. H. Glucose isomerase: functions, structures, and applications. *Applied Sciences* **12**, 428 (2022).

[49] Bush, K. & Bradford, P. A. Interplay between β-lactamases and new β-lactamase inhibitors. *Nature Reviews Microbiology* **17**, 295–306 (2019).

[50] Ma, L. *et al.* Ctx-m-14, a plasmid-mediated ctx-m type extended-spectrum β-lactamase isolated from escherichia coli. *Antimicrobial agents and chemotherapy* **46**, 1985–1988 (2002).

[51] Chen, Y., Shoichet, B. & Bonnet, R. Structure, function, and inhibition along the reaction coordinate of ctx-m β-lactamases. *Journal of the American Chemical Society* **127**, 5423–5434 (2005).

[52] Bonnet, R. Growing group of extended-spectrum β-lactamases: the ctx-m enzymes. *Antimicrobial agents and chemotherapy* **48**, 1–14 (2004).

[53] Dutour, C. *et al.* Ctx-m-1, ctx-m-3, and ctx-m-14 β-lactamases from enterobacteriaceae isolated in france. *Antimicrobial agents and chemotherapy* **46**, 534–537 (2002).

[54] He, D. *et al.* Residues distal to the active site contribute to enhanced catalytic activity of variant and hybrid β-lactamases derived from ctx-m-14 and ctx-m-15. *Antimicrobial agents and chemotherapy* **59**, 5976–5983 (2015).

[55] Tian, G.-B. *et al.* Characterization of ctx-m-140, a variant of ctx-m-14 extended-spectrum β-lactamase with decreased cephalosporin hydrolytic activity, from cephalosporin-resistant proteus mirabilis. *Antimicrobial Agents and Chemotherapy* **60**, 6121–6126 (2016).

[56] Fenn, T. D., Ringe, D. & Petsko, G. A. Xylose isomerase in substrate and inhibitor michaelis states: atomic resolution studies of a metal-mediated hydride shift. *Biochemistry* **43**, 6464–6474 (2004).

[57] Kovalevsky, A. Y. *et al.* Metal ion roles and the movement of hydrogen during reaction catalyzed by d-xylose isomerase: a joint x-ray and neutron diffraction study. *Structure* **18**, 688–699 (2010).

[58] Sanchez, S. & Smiley, K. L. Properties of d-xylose isomerase from streptomyces albus. *Applied Microbiology* **29**, 745–750 (1975).

[59] Cha, J. & Batt, C. A. Lowering the ph optimum of d-xylose isomerase: the effect of mutations of the negatively charged residues. *Molecules and cells* **8**, 374–382 (1998).

[60] Chauthaiwale, J. & Rao, M. Production and purification of extracellular d-xylose isomerase from an alkaliphilic, thermophilic bacillus sp. *Applied and environmental microbiology* **60**, 4495–4499 (1994).

[61] Toteva, M. M., Silvaggi, N. R., Allen, K. N. & Richard, J. P. Binding energy and catalysis by d-xylose isomerase: kinetic, product, and x-ray crystallographic analysis of enzyme-catalyzed isomerization of (r)-glyceraldehyde. *Biochemistry*

50, 10170–10181 (2011).

[62] Chanitnun, K. & Pinphanichakarn, P. Glucose (xylose) isomerase production by streptomyces sp. ch7 grown on agricultural residues. *Brazilian Journal of Microbiology* **43**, 1084–1093 (2012).

[63] Mehrabi, P. *et al.* The hare chip for efficient time-resolved serial synchrotron crystallography. *Journal of Synchrotron Radiation* **27**, 360–370 (2020).

[64] Masmaliyeva, R. C. & Murshudov, G. N. Analysis and validation of macromolecular b values. *Acta Crystallographica Section D: Structural Biology* **75**, 505–518 (2019).

[65] Pearce, N. M. & Gros, P. A method for intuitively extracting macromolecular dynamics from structural disorder. *Nature communications* **12**, 5493 (2021).

[66] Ginn, H. M. Torsion angles to map and visualize the conformational space of a protein. *Protein Science* **32**, e4608 (2023).

[67] Vagabond: bond-based parametrization reduces overfitting for refinement of proteins. *Acta Crystallographica Section D: Structural Biology* **77**, 424–437 (2021).

[68] Ren, Z. *et al.* Resolution of structural heterogeneity in dynamic crystallography. *Acta Crystallographica Section D: Biological Crystallography* **69**, 946–959 (2013).

[69] Pearce, N. M. *et al.* A multi-crystal method for extracting obscured crystallographic states from conventionally uninterpretable electron density. *Nature communications* **8**, 15123 (2017).

[70] Pearce, N. M., Krojer, T. & Von Delft, F. Proper modelling of ligand binding requires an ensemble of bound and unbound states. *Acta Crystallographica*

Section D: Structural Biology **73**, 256–266 (2017).

[71] Biener, G., Malla, T. N., Schwander, P. & Schmidt, M. Kinntrex: a neural network to unveil protein mechanisms from time-resolved x-ray crystallography. *IUCrJ* **11** (2024).

[72] Martin, R. W. & Zilm, K. W. Preparation of protein nanocrystals and their characterization by solid state nmr. *Journal of Magnetic Resonance* **165**, 162–174 (2003).

[73] Wiedorn, M. O. *et al.* Megahertz serial crystallography. *Nature communications* **9**, 4025 (2018).

[74] White, T. A. *et al.* Crystfel: a software suite for snapshot serial crystallography. *Journal of applied crystallography* **45**, 335–341 (2012).

[75] McCoy, A. J. *et al.* Phaser crystallographic software. *Journal of applied crystallography* **40**, 658–674 (2007).

[76] Adams, P. D. *et al.* Recent developments in the phenix software for automated crystallographic structure determination. *Journal of synchrotron radiation* **11**, 53–55 (2004).

[77] Emsley, P. & Cowtan, K. Coot: model-building tools for molecular graphics. *Acta crystallographica section D: biological crystallography* **60**, 2126–2132 (2004).

[78] Lohkamp, B., Scott, W. & Cowtan, K. Features and development of coot. *Acta Crystallographica. Section D: Biological Crystallography* **66** (2010).

[79] Schrödinger, LLC. The PyMOL molecular graphics system, version 1.8 (2015).

[80] Lang, P. T., Holton, J. M., Fraser, J. S. & Alber, T. Protein structural ensembles are revealed by redefining x-ray electron density noise. *Proceedings of the National Academy of Sciences* **111**, 237–242 (2014).

[81] Brookner, D. E. & Hekstra, D. R. Matchmaps: non-isomorphous difference maps for x-ray crystallography. *Journal of Applied Crystallography* (2024).

[82] Liebschner, D. *et al.* Polder maps: improving omit maps by excluding bulk solvent. *Acta Crystallographica Section D: Structural Biology* **73**, 148–157 (2017).

[83] Agirre, J. *et al.* The ccp4 suite: integrative software for macromolecular crystallography. *Acta Crystallographica Section D: Structural Biology* **79**, 449–461 (2023).

[84] Hill, J. A. *et al.* An ultraviolet-driven rescue pathway for oxidative stress to eye lens protein human gamma-d crystallin. *Communications Chemistry* **7**, 81 (2024).

[85] Wyckoff, H. *et al.* The structure of ribonuclease-s at 3.5 a resolution. *Journal of Biological Chemistry* **242**, 3984–3988 (1967).

[86] Kurisu, G., Sugimoto, A., Kai, Y. & Harada, S. A flow cell suitable for time-resolved x-ray crystallography by the laue method. *Journal of applied crystallography* **30**, 555–556 (1997).

[87] Helliwell, J. R. *et al.* Time-resolved and static-ensemble structural chemistry of hydroxymethylbilane synthase. *Faraday discussions* **122**, 131–144 (2003).

[88] Monteiro, D. C. *et al.* 3d-mixd: 3d-printed x-ray-compatible microfluidic devices for rapid, low-consumption serial synchrotron crystallography data collection in

flow. *IUCrJ* **7**, 207–219 (2020).

[89] Fuller, F. D. *et al.* Drop-on-demand sample delivery for studying biocatalysts in action at x-ray free-electron lasers. *Nature methods* **14**, 443–449 (2017).

[90] Sherrell, D. A. *et al.* A modular and compact portable mini-endstation for high-precision, high-speed fixed target serial crystallography at fel and synchrotron sources. *Journal of synchrotron radiation* **22**, 1372–1378 (2015).

[91] Makinen, M. W. & Fink, A. L. Reactivity and cryoenzymology of enzymes in the crystalline state. *Annual review of biophysics and bioengineering* **6**, 301–343 (1977).

[92] Moffat, K. Time-resolved macromolecular crystallography. *Annual review of biophysics and biophysical chemistry* **18**, 309–332 (1989).

[93] Fischer, M. Macromolecular room temperature crystallography. *Quarterly Reviews of Biophysics* **54**, e1 (2021).

[94] Martiel, I., Müller-Werkmeister, H. M. & Cohen, A. E. Strategies for sample delivery for femtosecond crystallography. *Acta Crystallographica Section D: Structural Biology* **75**, 160–177 (2019).

[95] Hekstra, D. R. *et al.* Electric-field-stimulated protein mechanics. *Nature* **540**, 400–405 (2016).

[96] Mo, F. & Ramsøskar, K. A sample cell for diffraction studies with control of temperature, relative humidity and applied electric field. *Journal of Applied Crystallography* **42**, 531–534 (2009).

[97] Saunders, L. K. *et al.* An electric field cell for performing in situ single-crystal synchrotron x-ray diffraction. *Journal of Applied Crystallography* **54**, 1349–1359

(2021).

[98] Garman, E. F. & Schneider, T. R. Macromolecular cryocrystallography. *Journal of Applied Crystallography* **30**, 211–237 (1997).

[99] Bowler, M. W., Montgomery, M. G., Leslie, A. G. & Walker, J. E. Reproducible improvements in order and diffraction limit of crystals of bovine mitochondrial f1-atpase by controlled dehydration. *Acta Crystallographica Section D: Biological Crystallography* **62**, 991–995 (2006).

[100] Sjögren, T. *et al.* Protein crystallography in a vapour stream: data collection, reaction initiation and intermediate trapping in naked hydrated protein crystals. *Journal of applied crystallography* **35**, 113–116 (2002).

[101] Sanchez-Weatherby, J. *et al.* Improving diffraction by humidity control: a novel device compatible with x-ray beamlines. *Acta Crystallographica Section D: Biological Crystallography* **65**, 1237–1246 (2009).

[102] Baba, S. *et al.* A temperature-controlled cold-gas humidifier and its application to protein crystals with the humid-air and glue-coating method. *Journal of Applied Crystallography* **52**, 699–705 (2019).

[103] Heras, B. & Martin, J. L. Post-crystallization treatments for improving diffraction quality of protein crystals. *Acta Crystallographica Section D: Biological Crystallography* **61**, 1173–1180 (2005).

[104] Sanchez-Weatherby, J. & Moraes, I. Crystal dehydration in membrane protein crystallography. *The Next Generation in Membrane Protein Structure Determination* 73–89 (2016).

[105] Awad, W., Svensson Birkedal, G., Thunnissen, M. M., Mani, K. & Logan, D. T. Improvements in the order, isotropy and electron density of glypican-1 crystals by controlled dehydration. *Acta Crystallographica Section D: Biological Crystallography* **69**, 2524–2533 (2013).

[106] Atakisi, H., Moreau, D. W. & Thorne, R. E. Effects of protein-crystal hydration and temperature on side-chain conformational heterogeneity in monoclinic lysozyme crystals. *Acta Crystallographica Section D: Structural Biology* **74**, 264–278 (2018).

[107] Klingl, S., Scherer, M., Stamminger, T. & Muller, Y. A. Controlled crystal dehydration triggers a space-group switch and shapes the tertiary structure of cytomegalovirus immediate-early 1 (ie1) protein. *Acta Crystallographica Section D: Biological Crystallography* **71**, 1493–1504 (2015).

[108] Heras, B. *et al.* Dehydration converts dsbg crystal diffraction from low to high resolution. *Structure* **11**, 139–145 (2003).

[109] Abergel, C. Spectacular improvement of x-ray diffraction through fast desiccation of protein crystals. *Acta Crystallographica Section D: Biological Crystallography* **60**, 1413–1416 (2004).

[110] Huang, Q. & Szebenyi, D. M. Improving diffraction resolution using a new dehydration method. *Acta Crystallographica Section F: Structural Biology Communications* **72**, 152–159 (2016).

[111] Warren, A. J., Axford, D. & Owen, R. L. Direct measurement of x-ray-induced heating of microcrystals. *Journal of Synchrotron Radiation* **26**, 991–997 (2019).

[112] Kriminski, S., Kazmierczak, M. & Thorne, R. E. Heat transfer from protein crystals: implications for flash-cooling and x-ray beam heating. *Acta*

Crystallographica Section D **59**, 697–708 (2003).

[113] Dickerson, J. L., McCubbin, P. T. N. & Garman, E. F. Raddose-xfel: femtosecond time-resolved dose estimates for macromolecular x-ray free-electron laser experiments. *Journal of Applied Crystallography* **53**, 549–560 (2020). URL http://scripts.iucr.org/cgi-bin/paper?S1600576720000643.

[114] Blumm, J. & Lindemann, A. Characterization of the thermophysical properties of molten polymers and liquids using the flash technique. *High Temp. High Press* **35**, 627 (2003).

[115] Inc., W. R. Mathematica, Version 14.2. URL https://www.wolfram.com/mathematica. Champaign, IL, 2024.
