# Supplementary material for: Probing the modulation of enzyme kinetics by multi-temperature, time-resolved serial crystallography
Source: Nat Commun. 2025 Jul 16;16:6553. doi: 10.1038/s41467-025-61631-2 (PMC12267849; doi:10.1038/s41467-025-61631-2)
Supplement: Supplementary file 2 — Reporting Summary [file 41467_2025_61631_MOESM2_ESM.pdf]

nature portfolio

Corresponding author(s): Eike C. Schulz, Pedram Mehrabi, Friedjof Tellkamp

Last updated by author(s): 2025/06/24

Reporting Summary

Nature Portfolio wishes to improve the reproducibility of the work that we publish. This form provides structure for consistency and transparency in reporting. For further information on Nature Portfolio policies, see our Editorial Policies and the Editorial Policy Checklist.

Statistics

For all statistical analyses, confirm that the following items are present in the figure legend, table legend, main text, or Methods section.

n/a	Confirmed	
☐	☒	The exact sample size (n) for each experimental group/condition, given as a discrete number and unit of measurement
☒	☐	A statement on whether measurements were taken from distinct samples or whether the same sample was measured repeatedly
☒	☐	The statistical test(s) used AND whether they are one- or two-sided<br>*Only common tests should be described solely by name; describe more complex techniques in the Methods section.*
☒	☐	A description of all covariates tested
☒	☐	A description of any assumptions or corrections, such as tests of normality and adjustment for multiple comparisons
☐	☒	A full description of the statistical parameters including central tendency (e.g. means) or other basic estimates (e.g. regression coefficient) AND variation (e.g. standard deviation) or associated estimates of uncertainty (e.g. confidence intervals)
☒	☐	For null hypothesis testing, the test statistic (e.g. F, t, r) with confidence intervals, effect sizes, degrees of freedom and P value noted<br>*Give P values as exact values whenever suitable.*
☒	☐	For Bayesian analysis, information on the choice of priors and Markov chain Monte Carlo settings
☒	☐	For hierarchical and complex designs, identification of the appropriate level for tests and full reporting of outcomes
☒	☐	Estimates of effect sizes (e.g. Cohen's d, Pearson's r), indicating how they were calculated

Our web collection on statistics for biologists contains articles on many of the points above.

Software and code

Policy information about availability of computer code

Data collection	Crystallographic data was collected using MxCube.
Data analysis	Serial diffraction data were processed using CrystFEL 0.10.1 with the XGANDALF indexing routine.<br>Refinement was carried out in the phenix suite 1.19 using phenix.refine and coot 0.8 for manual corrections to the model. POLDER maps were generated using phenix.polder. Molecular images were generated in PyMol3. Shifted inverse gamma distributions and Calpha RMSD were calculated as reported in the manuscript. ROPE data were calculated via ROPE version v0.1.6

For manuscripts utilizing custom algorithms or software that are central to the research but not yet described in published literature, software must be made available to editors and reviewers. We strongly encourage code deposition in a community repository (e.g. GitHub). See the Nature Portfolio guidelines for submitting code & software for further information.

Data

Policy information about availability of data

All manuscripts must include a data availability statement. This statement should provide the following information, where applicable:

- Accession codes, unique identifiers, or web links for publicly available datasets
- A description of any restrictions on data availability
- For clinical datasets or third party data, please ensure that the statement adheres to our policy

All crystallographic data have been deposited in the protein data bank under https://www.rcsb.org/.

Data for the XI humidity series have been deposited under the accession numbers: 9G5N, 9G5S, 9G5W, 9G5X, 9G61, 9G6L, 9G6M, 9G6N, 9G6O, 9G6P
Data for the SSX datasets of CTXM-14 have been deposited under the accession numbers: 9G7V, 9G7W, 9G7X, 9G7Y, 9G7Z, 9G80, 9G81, 9G82

Human research participants

Policy information about studies involving human research participants and Sex and Gender in Research.

Reporting on sex and gender	n/a
Population characteristics	n/a
Recruitment	n/a
Ethics oversight	n/a

Note that full information on the approval of the study protocol must also be provided in the manuscript.

Field-specific reporting

Please select the one below that is the best fit for your research. If you are not sure, read the appropriate sections before making your selection.

[x] Life sciences [ ] Behavioural & social sciences [ ] Ecological, evolutionary & environmental sciences

For a reference copy of the document with all sections, see nature.com/documents/nr-reporting-summary-flat.pdf

Life sciences study design

All studies must disclose on these points even when the disclosure is negative.

Sample size	The sample size was determined by the number of integrated diffraction images, the number of integrated reflections, respectively.
Data exclusions	No data were excluded from the analysis
Replication	In SSX data several crystals were measured and merged for their respective structures.
Randomization	5% of the reflections were excluded for Rfree value calculation
Blinding	No blinding was conducted

Reporting for specific materials, systems and methods

We require information from authors about some types of materials, experimental systems and methods used in many studies. Here, indicate whether each material, system or method listed is relevant to your study. If you are not sure if a list item applies to your research, read the appropriate section before selecting a response.

Materials & experimental systems

n/a	Involved in the study	
[x]	[ ]	Antibodies
[x]	[ ]	Eukaryotic cell lines
[x]	[ ]	Palaeontology and archaeology
[x]	[ ]	Animals and other organisms
[x]	[ ]	Clinical data
[x]	[ ]	Dual use research of concern

Methods

n/a	Involved in the study	
[x]	[ ]	ChIP-seq
[x]	[ ]	Flow cytometry
[x]	[ ]	MRI-based neuroimaging
